# Exploration of the intelligent control system of autonomous vehicles based on edge computing

**DOI:** 10.1371/journal.pone.0281294

**Published:** 2023-02-02

**Authors:** Guo Ming

**Affiliations:** School of Automotive Engineering, Wuhan University of Technology, Wuhan City, PR. China; Zonguldak Bülent Ecevit University: Zonguldak Bulent Ecevit Universitesi, TURKEY

## Abstract

The development of science and technology continues to promote the progress of society. The current intelligence and automation technology has become widely used in society. To this end, this study proposes a vehicle intelligent control system based on edge computing and deep learning to promote the far-reaching development of intelligent technology and automation technology. First, control algorithms are used to design a switch control strategy combining accelerator and brake. Second, a fuzzy control algorithm based on vehicle tracking and trajectory deviation is designed to enhance the vehicle’s stability during steering. A Convolutional Neural Network (CNN) is used to recognize the car’s surroundings as it drives. In addition, accelerator and brake controllers and vehicle tracking and trajectory deviation controllers are connected to the vehicle’s wiring. Then, the data transmission function based on edge computing is applied to the vehicle’s intelligent control system. Finally, trajectory tracking and emergency braking experiments are carried out on the control system to verify the practicability and reliability of the method and the effectiveness of CNN. The simulation experiments are carried out on two states of medium speed and high speed to verify the effectiveness of the longitudinal anti-collision system of the test vehicle when the target vehicle suddenly decelerates. The results demonstrate that the driving speed of the experimental vehicle is set to 50km/h, the distance between the experimental vehicle and the target vehicle is 40m, and the target vehicle in front drives at a constant speed of 50km/h. The target vehicle in front of the car suddenly decelerates in 5 seconds, and the speed drops to 0 after 5 seconds. The actual distance between the experimental vehicle and the target vehicle is very close to the expected safe space, and the experimental vehicle is in a safe state during this process. When the experimental vehicle starts to decelerate, the experimental vehicle adopts emergency deceleration to ensure a safe distance between the two vehicles. At this time, the car enters the second-level early warning state, but driving safety can still be guaranteed. It is advisable to maintain low-speed emergency braking in this state. This study provides creative research ideas for the follow-up research on the intelligent control system of uncrewed vehicles and contributes to the development of intelligence and automation technology.

## Introduction

Artificial intelligence (AI) technology has been deeply integrated into people’s daily lives with the continuous development of science and technology. Some smart technologies have been implanted into ordinary cars, such as automatic parking and active braking technology. Driverless cars obtain and identify the vehicle’s surrounding environment by installing various sensors and then conduct correct analysis and judgment through the corresponding intelligent control system. It prompts the intelligent design to make proper decisions and correct controls. The movement of the vehicle realizes actual uncrewed driving [1]. The essential difference between driverless and ordinary cars lies in the different degrees of intelligence. Uncrewed vehicles can realize all-around control of the driving process of the vehicle and independently complete operations such as acceleration and steering [2]. The motion control of uncrewed vehicles is mainly reflected in the vertical and horizontal intelligent control systems. The longitudinal control system refers to the management of the acceleration and deceleration of the car; the lateral control system refers to the control of the car’s steering operation and path tracking [3]. The basis for realizing driverless cars is to ensure driving safety, so the car’s anti-collision system is fundamental [4]. The automobile anti-collision system mainly relies on onboard sensors to collect relevant information on automobile driving to realize real-time detection of safe driving. In an emergency, the collision avoidance system can send an early warning to the intelligent control system. The smart control system can take corresponding measures through the early warning information to ensure the safety of the people in the vehicle to a certain extent and reduce the frequency and loss of traffic accidents [5]. It can be seen that society’s demand for AI technology and automation technology is getting increasingly high under the continuous development and improvement of uncrewed driving technology. Combining deep learning technology with edge computing technology to meet the needs of society has improved the application effect of AI technology. Still, the current application of this technology in autonomous driving is not sufficiently mature. Therefore, further research is needed to provide support for its development.

At present, many researchers have researched uncrewed vehicles. Yu et al. (2018) proposed a path-planning method for the navigation control system of uncrewed electric buses. They used the Dijkstra algorithm based on the ArcGIS analysis tool, improving the driving efficiency and reducing the complexity of intelligent system calculations [6]. Wells et al. (2020) developed an algorithm for planning corresponding driving trajectories between two points specified on a map. This approach will make a practical contribution to navigation systems for self-driving cars [7]. Yağ and Altan (2022) proposed a predictive control algorithm based on an adaptive predictive model to improve trajectory tracking accuracy and designed a lateral motion control strategy. Finally, they utilized MATLAB/Simulink and CarSim co-simulation. The results showed that the control strategy improves the driving stability of the uncrewed racing car and the trajectory tracking accuracy conditions under the limit state [8]. Lyons (2022) presented a dynamic obstacle detection and tracking method based on multi-feature fusion and a dynamic obstacle recognition method based on spatiotemporal feature vectors. Firstly, the pulse width characteristics of obstacle echoes were considered to improve the accuracy of obstacle detection and tracking based on the geometric attributes of dynamic obstacles. Secondly, the space-time feature vector was constructed based on the obstacle time dimension and space dimension information. Then, the support vector machine method was adopted to recognize dynamic obstacles to improve the accuracy of obstacle recognition. Finally, the accuracy and effectiveness of the proposed method were verified by a real vehicle test [9]. Tan et al. (2022) explored the application of deep learning in driverless cars in response to the urgent need of contemporary society to develop driverless vehicles. First, the authors discussed driverless cars’ working principles and architectural design. Then, the authors applied unsupervised learning to cluster the data in each layer and supervised learning to adjust the relationship between each layer based on the basic concepts. Besides, it was applied to the deceleration horn and acceleration and deceleration control in driving [10]. It can be seen that the actual development of uncrewed driving technology is not ideal. Therefore, it is necessary to improve the degree of perfection of uncrewed driving technology by improving the application method of AI technology.

In summary, although many scholars have researched uncrewed driving technology, its development status is not perfect. Therefore, this study introduces deep learning technology to promote the development of uncrewed driving technology. Besides, edge computing technology is discussed. Finally, incremental Proportion Integration Differentiation (PID) is adopted to design the throttle system to reduce the error rate of control. Meanwhile, the fuzzy algorithm is employed to design the braking system to improve the stability of control based on reducing the amount of calculation. In addition, the path tracking algorithm and fuzzy control algorithm based on the course deviation is used to design the lateral control system to effectively improve the stability of the vehicle lateral control system and trajectory tracking. The innovation of this study lies in the efficient integration of deep learning technology and edge computing technology, highlighting both advantages and optimizing their application effect. The research reported here provides technical support for the development of AI technology and facilitates the development of driverless technology.

## Method

### Basic framework and technical support for driverless cars

**Fig 1:**
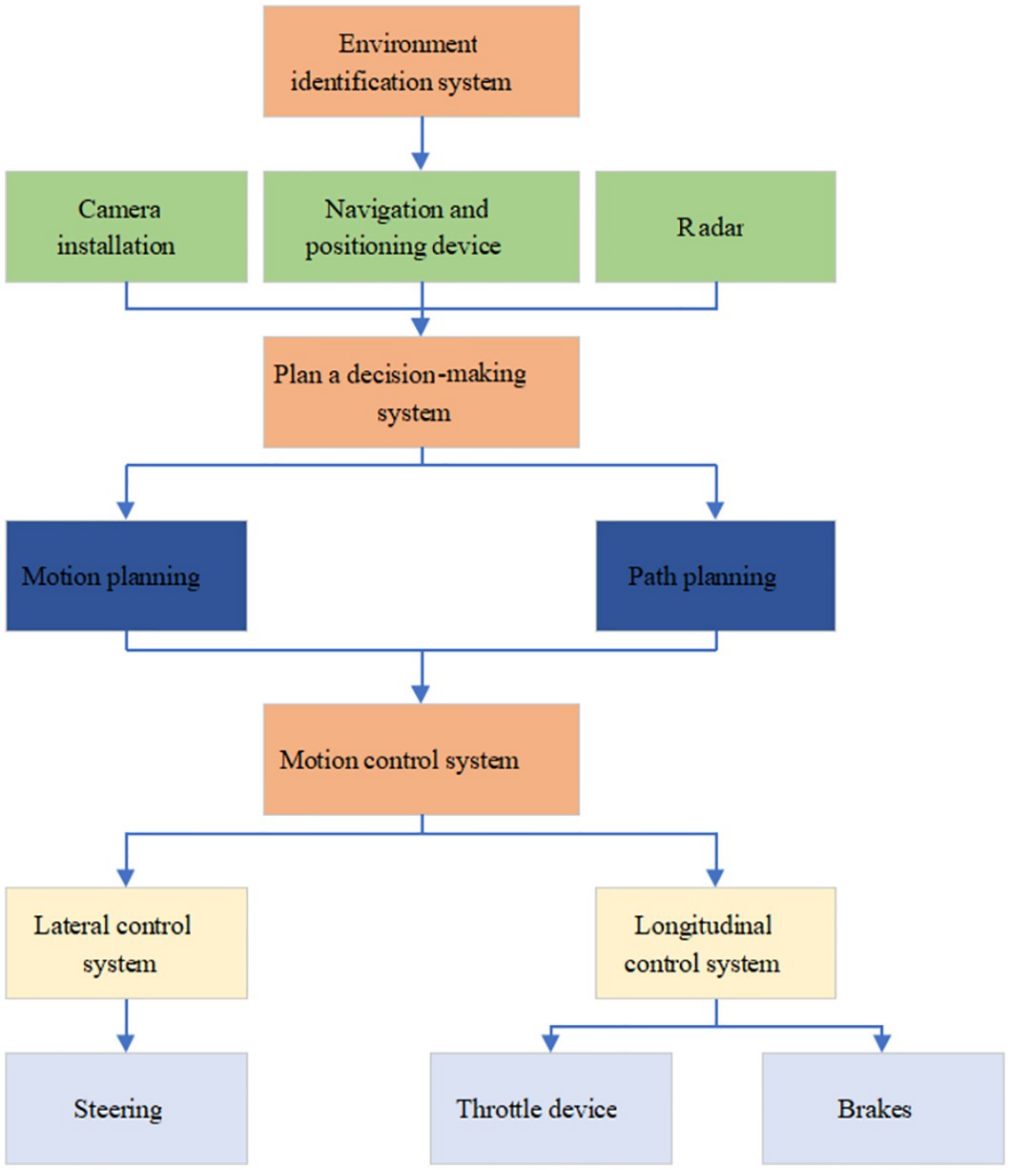
Intelligent autonomous vehicle system.

The overall driverless vehicle framework includes an environment perception system [11] to recognize the surrounding environment, a planning decision tree system [12] to plan the driving route, and a motion control system [13] to control the behavior of the vehicle. Fig 1 presents the specific architecture.

Autonomous vehicles depend on positioning and navigation technology [14], information fusion technology [15], and wire-based transformation technology [16]. The car navigation and positioning technology locates the vehicle’s position and plans the vehicle’s driving route simultaneously, which is the basis of the control of the driverless car.

### PID control algorithm

**Fig 2:**
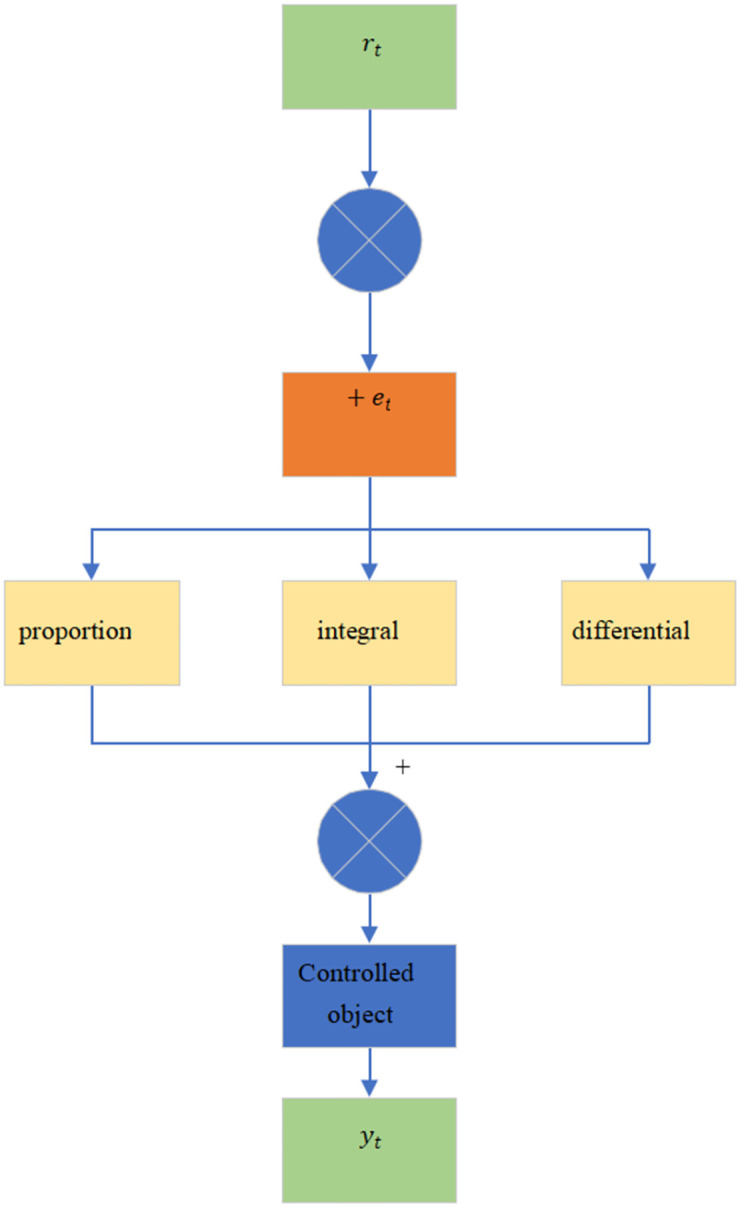
Fundamentals of the PID control algorithm.

The PID control algorithm is a relatively common and classic control method. It is mainly composed of three parts: proportional (P), integral (I), and differential (D). The linear relationship is obtained by calculating the target control amount through the difference between the input and output values of the input system. Fig 2 reveals the primary rationale.

In Fig 2, *r*(*t*) denotes the system input value, *y*(*t*) represents the system output value, and *e*(*t*) stands for the deviation between the input and output values. The obtained variation is input into the controller. The corresponding control quantity *u*(*t*) can be obtained through the combined operation. Then, the correction of the output value of the controlled object can be realized. The PID operation can be described as:

$$u\left(t\right)=K_{p}\left[e\left(t\right)+\frac{1}{T_{i}}\int_{0}^{t}e\left(t\right)+T_{d}\frac{de\left(t\right)}{dt}\right]$$ (1)

$$e\left(t\right)=r\left(t\right)-y\left(t\right)$$ (2)

where *K*_*p*_ represents the proportional coefficient; *T*_*i*_ denotes the time constant corresponding to the integral; *T*_*d*_ refers to the differential time constant.

The PID algorithm is mainly divided into incremental and positional [17]. Eq (3) describes the position-mode PID.


$$u\left(t\right)=K_{p}\left[e\left(t\right)+\frac{1}{T_{i}}\Sigma_{i=0}^{t}e\left(i\right)T+T_{d}\frac{e\left(t\right)-e\left(t-1\right)}{T}\right]$$ (3)

It can also be expressed as Eq (4).


$$u\left(t\right)=K_{p}e\left(t\right)+K_{i}\Sigma_{i=0}^{t}e\left(i\right)+K_{d}\left[e\left(t\right)-e\left(t-1\right)\right]$$ (4)

In Eq (4), $K_{i}=K_{p}\frac{T}{T_{i}}$, and $K_{d}=K_{p}\frac{T_{d}}{T}$; *K*_*p*_ represents the proportional coefficient; *K*_*i*_ denotes the integral coefficient; *K*_*d*_ refers to the differential coefficient; *T* stands for the sampling time serial number.

The integral coefficient term in Eq (4) signifies the accumulated value of the deviation, and the calculation amount increases gradually with the increase of time. The incremental PID algorithm takes the control amount as the output. The increment represents the amount that needs to be raised at the current moment and the previous moment, as presented in Eq (5).


$$\Delta u\left(t\right)=u\left(t\right)-u\left(t-1\right)=K_{p}\left\{e\left(t\right)-e\left(t-1\right)+K_{i}e\left(t\right)+K_{d}\left[e\left(t\right)-2e\left(t-1\right)+e\left(t-2\right)\right]\right\}$$ (5)

In Eq (5), Δ*u*(*t*) refers to the control output of the incremental PID control algorithm.

### Intelligent control system for autonomous vehicles based on edge computing

#### Driverless cars based on edge computing

**Fig 3:**
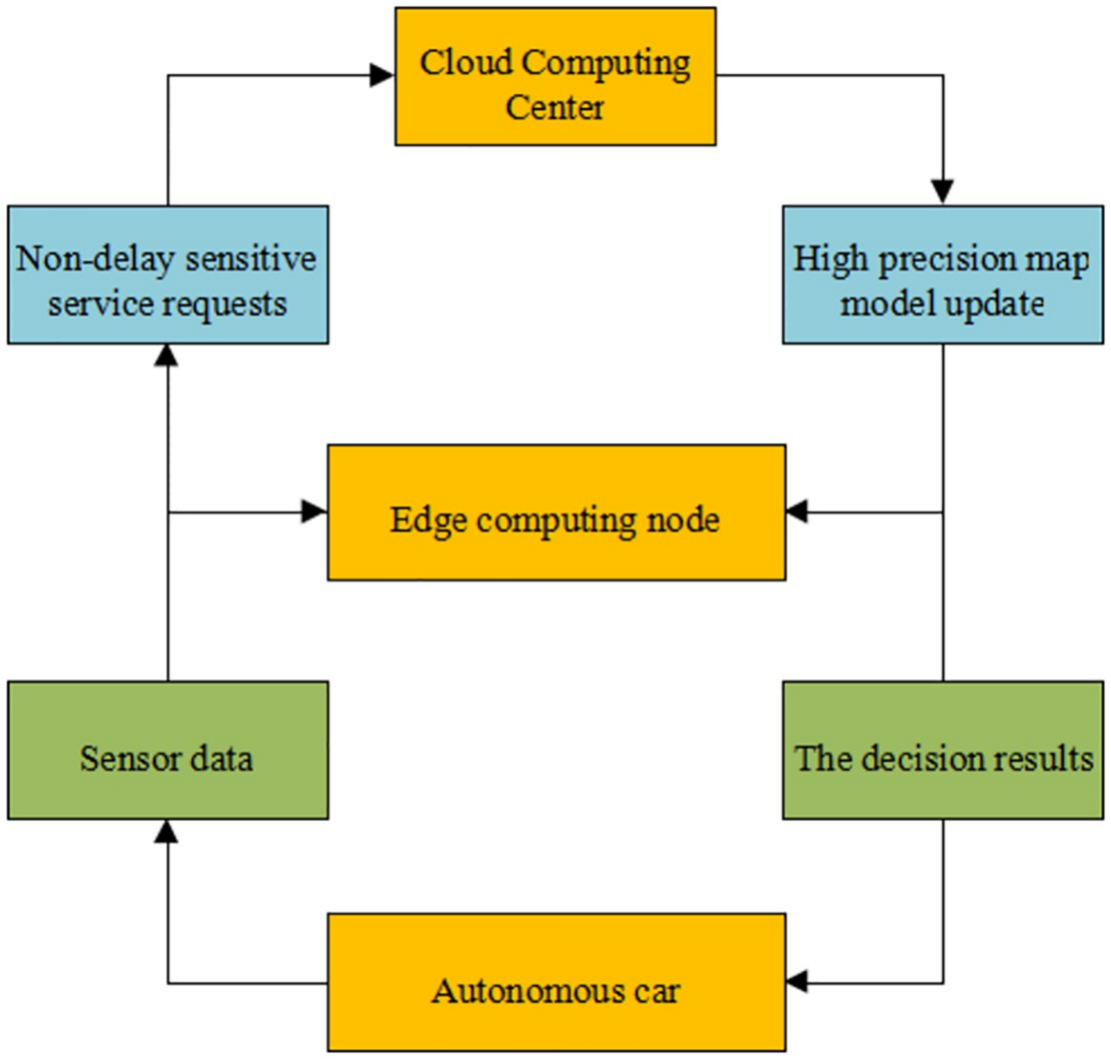
Architecture of autonomous vehicles based on edge computing.

With the rapid development of science, technology, and AI, people’s expectations of self-driving cars are getting even higher. However, because the intelligent control system of self-driving vehicles has an enormous computational task, the computational performance of current onboard computers is inadequate to meet the needs of self-driving cars. At the same time, cost control is necessary for improving computing power, which is a significant challenge for the industrialization of autonomous vehicles. Researchers have proposed a cloud-based self-driving car system with the help of connected vehicle technology and cloud computing. They introduce edge computing into the intelligent control system of the car to balance the computational load of each task of the driving vehicle and reduce the task load. Fig 3 reveals the architecture of self-driving cars based on edge computing.

Although the edge computing architecture in Fig 3 can solve many problems, there are still specific problems in data transmission between cars and edge nodes due to a large amount of data collection and processing for autonomous vehicles. The Vehicle-to-edge Data Transmission (VDT) problem is optimized to ensure autonomous vehicles’ safety, practicability, and stability. Besides, two heuristic algorithms are proposed to find an excellent solution in a limited time to furnish the VDT problem with a practical optimization strategy.

**Fig 4:**
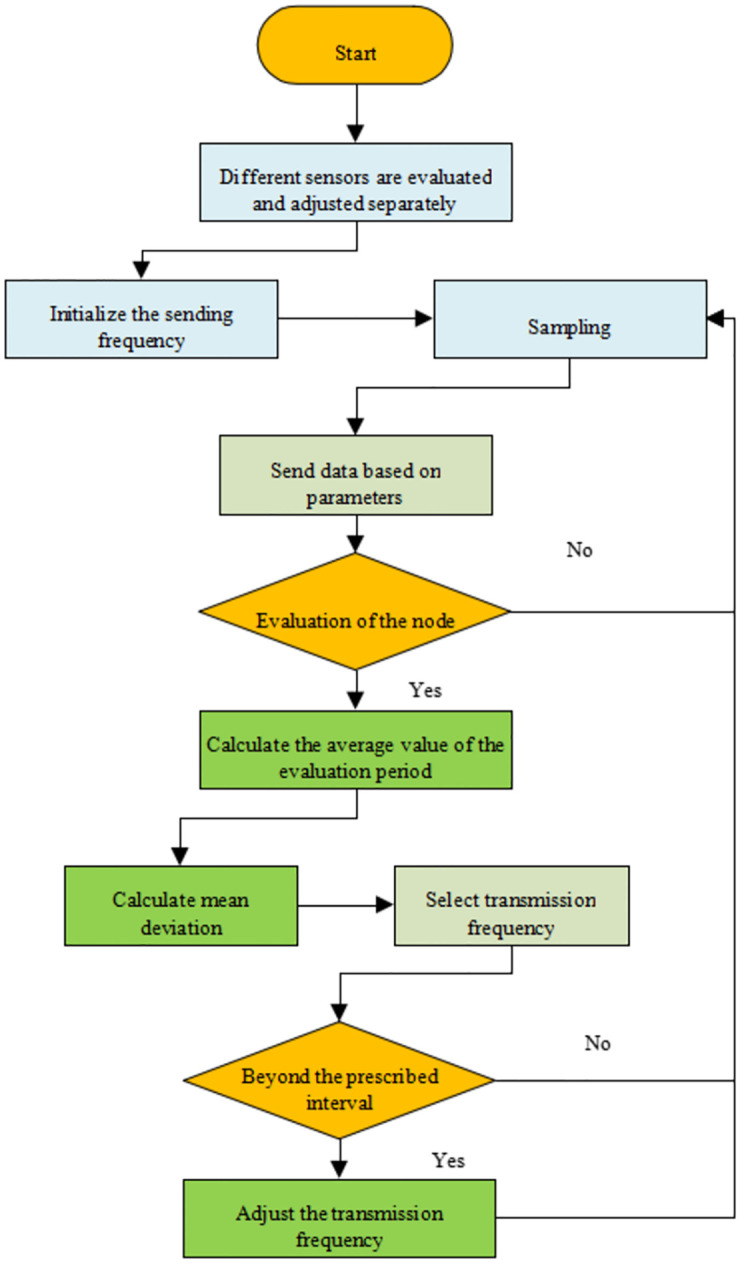
Flow of the deviation detection algorithm.

The deviation detection algorithm can flexibly use various aggregation functions and optimize part of the data in real-time at any period, effectively reducing the algorithm’s complexity. The deviation detection algorithm detects and evaluates the data transmission in the evaluation period and adjusts the correlation coefficient of the sensor data transmission in one period. Fig 4 is the flowchart of the deviation detection algorithm.

Fig 4 shows the basic design idea and the overall framework of the deviation detection algorithm. The mean value corresponding to the two types of sensor data needs to be calculated in each evaluation period.

**Fig 5:**
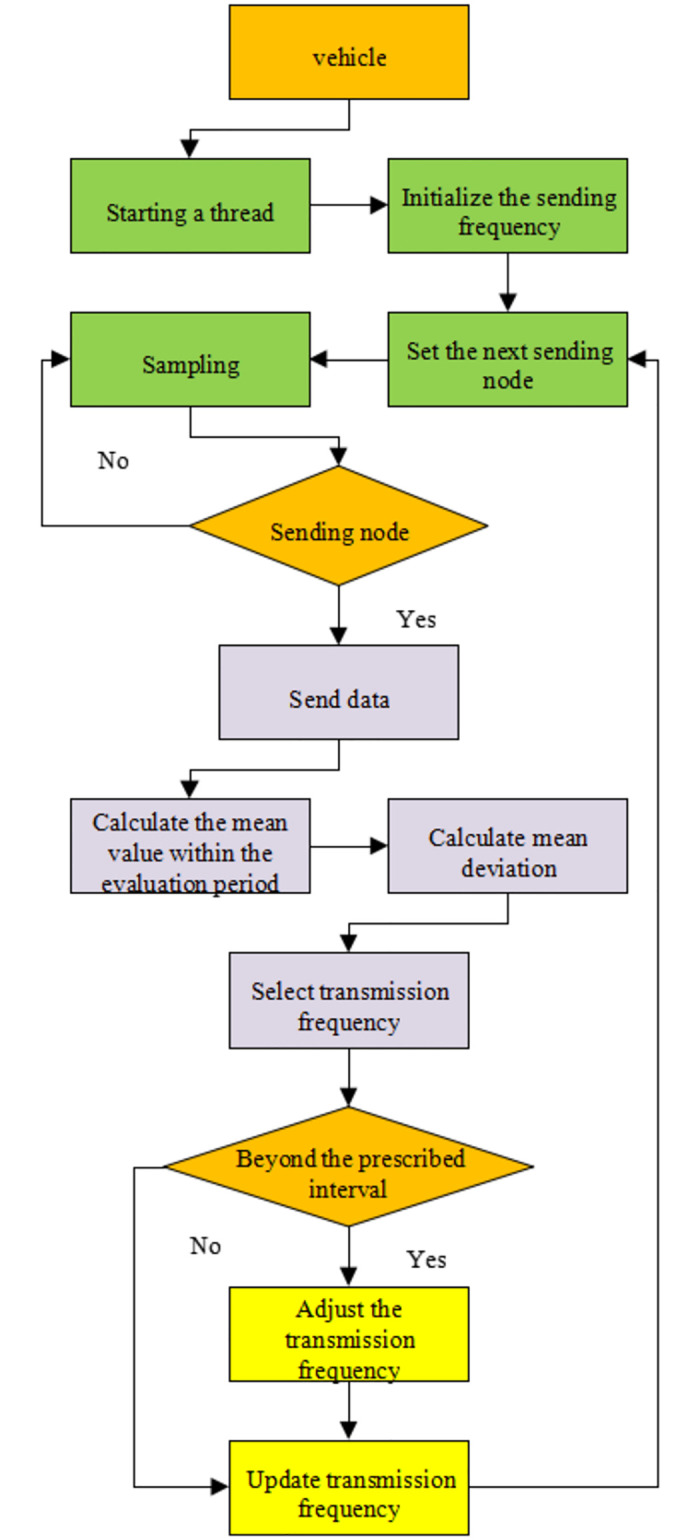
Procedures of the greedy algorithm.

The deviation detection algorithm takes all the cars in a specific road section as a whole and evaluates and adjusts the entire in a fixed period. The basic design idea of the greedy algorithm is to obtain the local optimal solution as much as possible under the premise of ensuring the overall information quality. Then, it obtains the optimization result close to the global optimal solution based on the local optimal solution of all vehicles. Fig 5 presents the overall design framework of the greedy algorithm.

Each type of sensor data of each autonomous vehicle needs to be collected. The framework is used to process the data separately in a multi-threaded manner. In other words, the global framework of the deviation detection algorithm is disassembled into a single-vehicle-oriented process.

### Design of the longitudinal control system

#### Throttle controller design

The longitudinal driving process of the car is easily affected by the resistance. The driving force of the forward driving of the vehicle must be greater than or equal to the sum of the resistance. Hence, the design of the accelerator control system must be based on the longitudinal dynamic model of the car [18]. The control object of the accelerator control system designed here is the stroke amount simulated by the accelerator pedal, and the speed deviation is selected as the input amount. The various factors affecting the speed during the driving process are calculated using the formula to confirm the corresponding speed influence amount. Then, the final speed influence amount is obtained by adding up, and the initial speed deviation and the speed influence amount of the resistance are used as the final speed deviation *e*(*t*). Here, the incremental PID algorithm is used to design the throttle control system to ensure the stable control of the vehicle and reduce the amount of calculation and error rate.

Because the vehicle will encounter various complex situations during the driving process, the ordinary incremental PID algorithm will have the problems of inaccurate control and large error to a certain extent. Therefore, the incremental PID is optimized by introducing the four-point central difference method. This optimization can enhance the applicability of the incremental PID algorithm to the environment in the driving process of the car. Since the differential term deviation greatly influences the control variable in the ordinary algorithm, the problem of adjustment failure or delay will occur when the control variable exceeds the upper and lower limits. The differential term is corrected by the four-point central difference method to suppress the above phenomenon. Eq (6) indicates the corresponding mathematical expression.


$$\bar{e}\left(t\right)=\frac{\left[e\left(t\right)+e\left(t-1\right)+e\left(t-2\right)+\left(t-3\right)\right]}{4}$$ (6)

When time is considered, the differential term is approximated by a weighted summation.


$$\frac{T_{d}*\Delta e}{T}=\frac{T_{d}}{4}\left[\frac{e\left(t\right)-\bar{e}\left(t\right)}{1.5T}+\frac{e\left(t-1\right)-\bar{e}\left(t\right)}{0.5T}+\frac{\bar{e}\left(t\right)-e\left(t-2\right)}{0.5T}+\frac{\bar{e}\left(t\right)-e\left(t-3\right)}{1.5T}\right]=\frac{T_{d}}{6T}\left[e\left(t\right)+3e\left(t-1\right)-e\left(t-2\right)-3e\left(t-3\right)\right]$$ (7)

The parameters of the PID control system are determined through the adjustment experience of parameters and the test of a large number of experimental vehicles: *K*_*p*_ = 2.5, *K*_*i*_ = 0.03, and *K*_*d*_ = 2.5.

### Design of the brake controller

The design of the brake controller using the common PID algorithm will be affected by frequent control. Frequent trampling of the brake pedal will lead to aggravated vibration of the vehicle and reduce comfort. Fuzzy control shows high stability, so the fuzzy control algorithm is used to design the braking controller [19], divided into the following four steps.

**Fig 6:**
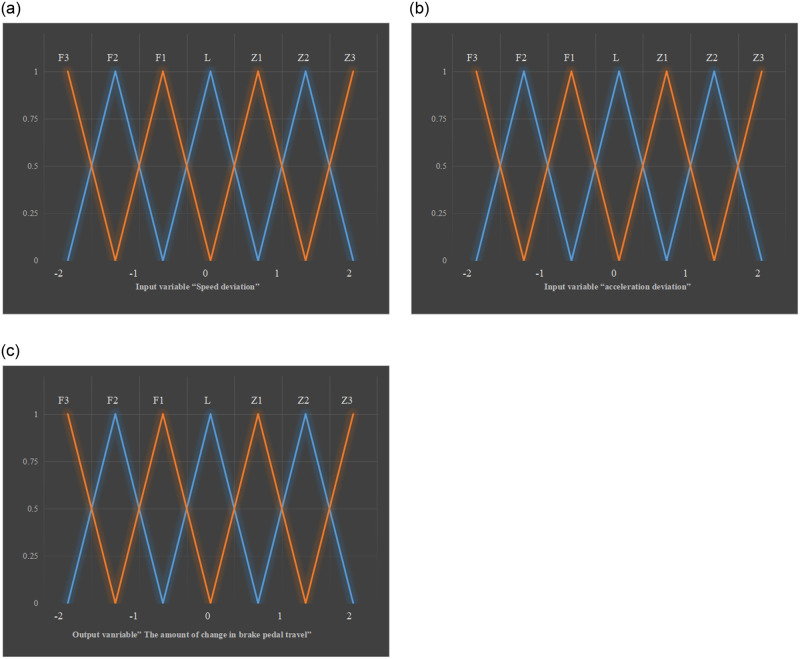
Membership functions of input and output variables (a) velocity deviation (b) acceleration deviation (c) brake pedal stroke change.

1. Determination of the input variable and output variable
  In the process of designing the fuzzy brake controller, the input variables select speed deviation *e*_*v*_ and acceleration deviation *e*_*a*_; the variation of the brake pedal stroke Δ*s* represents the output variable [20]. If the pedal does not change, the pedal stroke is 0%; the maximum pedal stroke is 100%, meaning that the brake pedal is fully depressed. The stroke change Δ*s* represents the difference between the current time and the previous time. A positive Δ*s* means pressing down on the brake pedal, and a negative Δ*s* means the pedal rebounds.
2. Variable fuzzification
  The value range of the speed deviation *e*_*v*_ is [-1, 1]; the unit is m/s; the value range corresponding to the acceleration deviation *e*_*a*_ is [-0.1, 0.1], and the unit is *m*/*s*^2^. The fuzzy set domain corresponding to acceleration deviation and velocity deviation is [-2, 2]; the value range of the output variable brake pedal stroke change Δ*s* is [-60%, 60%]; the fuzzy set domain is [-3, 3].
  The fuzzy subset of two input variables *e*_*v*_ and *e*_*a*_ is F3, F2, F1, L, Z1, Z2, and Z3. These elements represent the expected velocity relative to the actual velocity or the expected acceleration relative to the actual acceleration: minimum, very small, small, moderate, large, very large, and maximum. The output variable fuzzy set is F3, F2, F1, L, Z1, Z2, and Z3. They means that the brake pedal is lifted a lot, lifted by an appropriate amount, lifted a little, kept unchanged, stepped on a little, stepped on an appropriate amount, and stepped on a lot.
  The available set corresponding to fuzzy subsets of input variables and output variables is expressed as:
$$\left\{e_{v},\;e_{a}\right\}=\left\{\text{F}3,\text{F}2,\text{F}1,\text{Z}1,\text{Z}2,\text{Z}3\right\}$$ (8)
$$\Delta s=\left\{\text{F}3,\text{F}2,\text{F}1,\text{Z}1,\text{Z}2,\text{Z}3\right\}$$ (9)
  A triangular function is selected as the membership function of input and output variables [21], as shown in Fig 6.
3. Construction of fuzzy control rules
  Table 1 illustrates the fuzzy control rules formulated after analyzing the driver’s braking behavior during driving.
  In Table 1, the column corresponds to the velocity deviation *e*_*v*_, and the row corresponds to the acceleration deviation *e*_*a*_.
4. Clarification of output variables
  The output obtained by fuzzy rules is fuzzy not the final output. The corresponding fuzzy reasoning and clear calculation are carried out by the area centroid method. The brake control system can realize the automatic control of the brake system by controlling the brake pedal through the output quantity.

**Table 1:** Fuzzy control rules.

Brake pedal stroke change		Acceleration deviation *e*_*a*_						
		F3	F2	F1	L	Z1	Z2	Z3
Speed deviation *e*_*v*_	F3	Z3	Z3	Z2	Z2	Z1	Z1	L
	F2	Z3	Z2	Z2	Z1	Z1	L	F1
	F1	Z2	Z2	Z1	Z1	L	F1	F1
	L	Z2	Z1	Z1	L	F1	F1	F2
	Z1	Z1	Z1	L	F1	F1	F2	F2
	Z2	Z1	L	F1	F1	F2	F2	F3
	Z3	L	F1	F1	F2	F2	F3	F3

### Throttle and brake control switching

During normal driving, the car must switch between the accelerator and the brake frequently. The control system of the driverless car needs to switch the accelerator and brake smoothly according to the current road’s actual situation to realize the car’s stable control in the longitudinal direction.

The switching between the accelerator and the brake of the driverless car is mainly based on the current controller state and speed deviation *e*_*v*_ to determine the switching mode. The specific situations are divided into the following three situations.

1. The accelerator and brake controllers are not controlled; that is to say, neither the accelerator nor the brake is stepped on. When *e*_*v*_ > 0, the throttle controller presses down on the car to accelerate its motion. When *e*_*v*_ < 0, the brake controller stepped on the car to decelerate.
2. When the accelerator controller is in the acceleration state, the simulated stroke of the accelerator pedal is regulated through *e*_*v*_ to control acceleration and deceleration. When the simulated stroke of the accelerator pedal is 0, *e*_*v*_ < 0, and the brake control is switched to perform deceleration action.
3. When the brake controller is in the control state, the simulated stroke of the accelerator pedal is regulated through *e*_*v*_ to control acceleration and deceleration. When the simulated stroke of the brake pedal is 0, *e*_*v*_ > 0. The target speed is not reached at this time, so the accelerator control is switched to perform acceleration action.

A transition area is set before the throttle and brake control to efficiently control the stable driving of the vehicle. A threshold *h* is set 0.5*m*/*s* for *e*_*v*_. When |*e*_*v*_| < 0.5, the mode is not switched; when |*e*_*v*_| > 0.5, the mode is switched according to the actual situation.

### Design of the lateral control system

#### Path tracking algorithm based on trajectory deviation

The path tracking algorithm based on heading deviation plays a critical part in applying uncrewed vehicle path planning. It mainly acts on the lateral control system of the driverless vehicle to correct the deviation value between the actual heading of the vehicle and the target heading in real time, continuously reducing the deviation value and finally making the two consistent [22]. The vehicle heading angle change in the unit period during the turning process is calculated according to Eq (10).


$$\delta_{f}=\frac{\theta }{k}$$ (10)

In Eq (10), *δ*_*f*_ signifies the deflection angle of the front wheel of the car; *θ* stands for the steering wheel angle; *k* represents the ratio of the deflection angle of the steering wheel to the front wheel.

The turning radius of the car is *R*, which calculated through Eq (11).


$$R=\frac{a+b}{\text{tan}\left(\delta_{f}*\frac{\pi }{180}\right)}$$ (11)

Eq (12) indicates the change in heading angle from time *t* to time *t* − 1 in a unit period.


$$\beta_{t}=\frac{l_{t}}{R}=\frac{l_{t}*\text{tan}\left(\delta_{f}*\frac{\pi }{180}\right)}{a+b}$$ (12)

In Eq (12), *l*_*t*_ denotes the path that the car travels from time *t* − 1 to time *t*.

**Fig 7:**
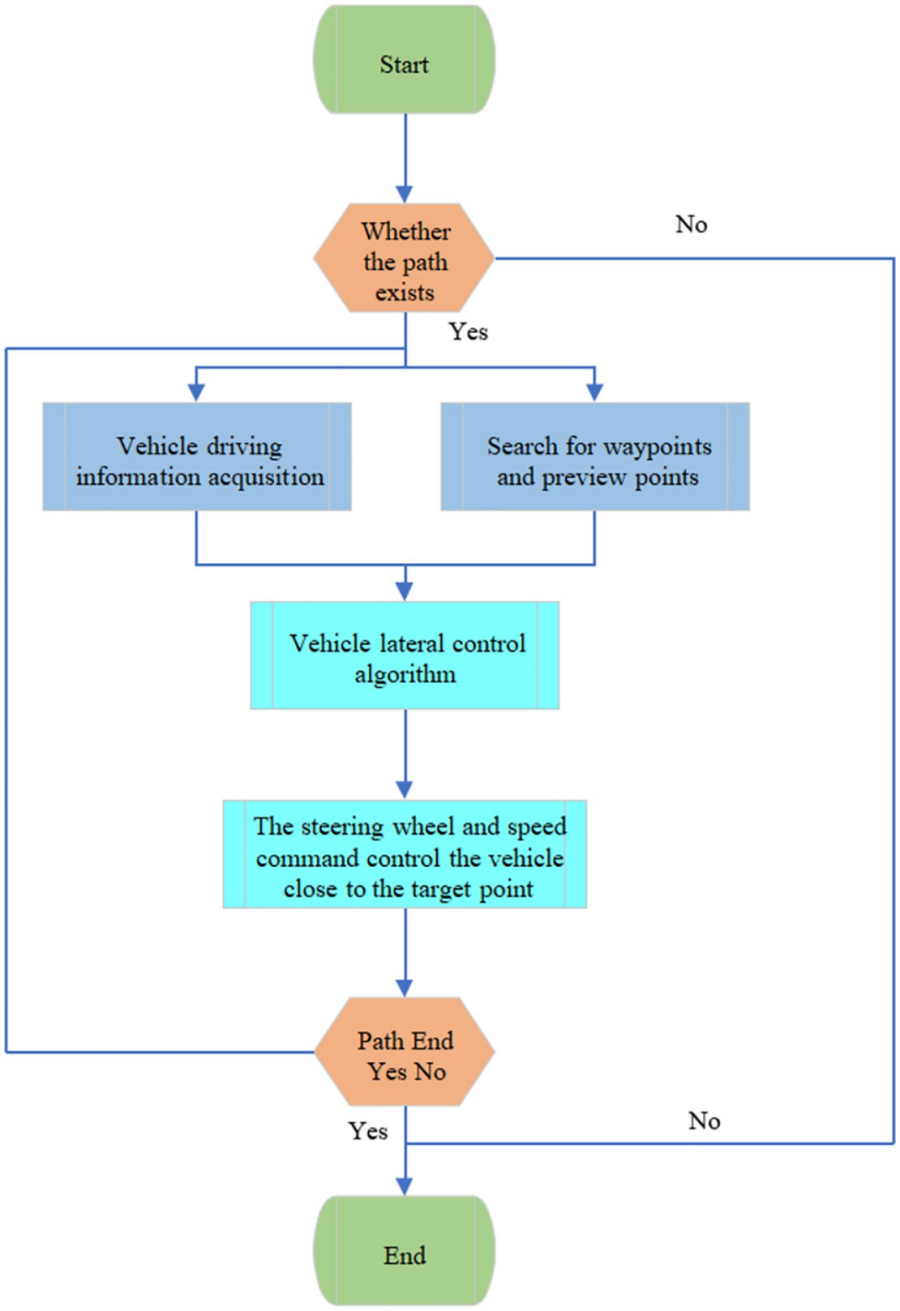
Basic flow of the path-tracing algorithm.

Fig 7 reveals the flow of the path tracking algorithm based on heading deviation.

### Design of the steering controller

A fuzzy control algorithm based on heading deviation and path tracking algorithm is proposed to improve the accuracy and comfort of lateral control without increasing the amount of calculation. The algorithm is implemented through the Matlab fuzzy logic toolbox (Fuzzy Logic Designer). The design of the binning controller for the algorithm is as follows.

**Fig 8:**
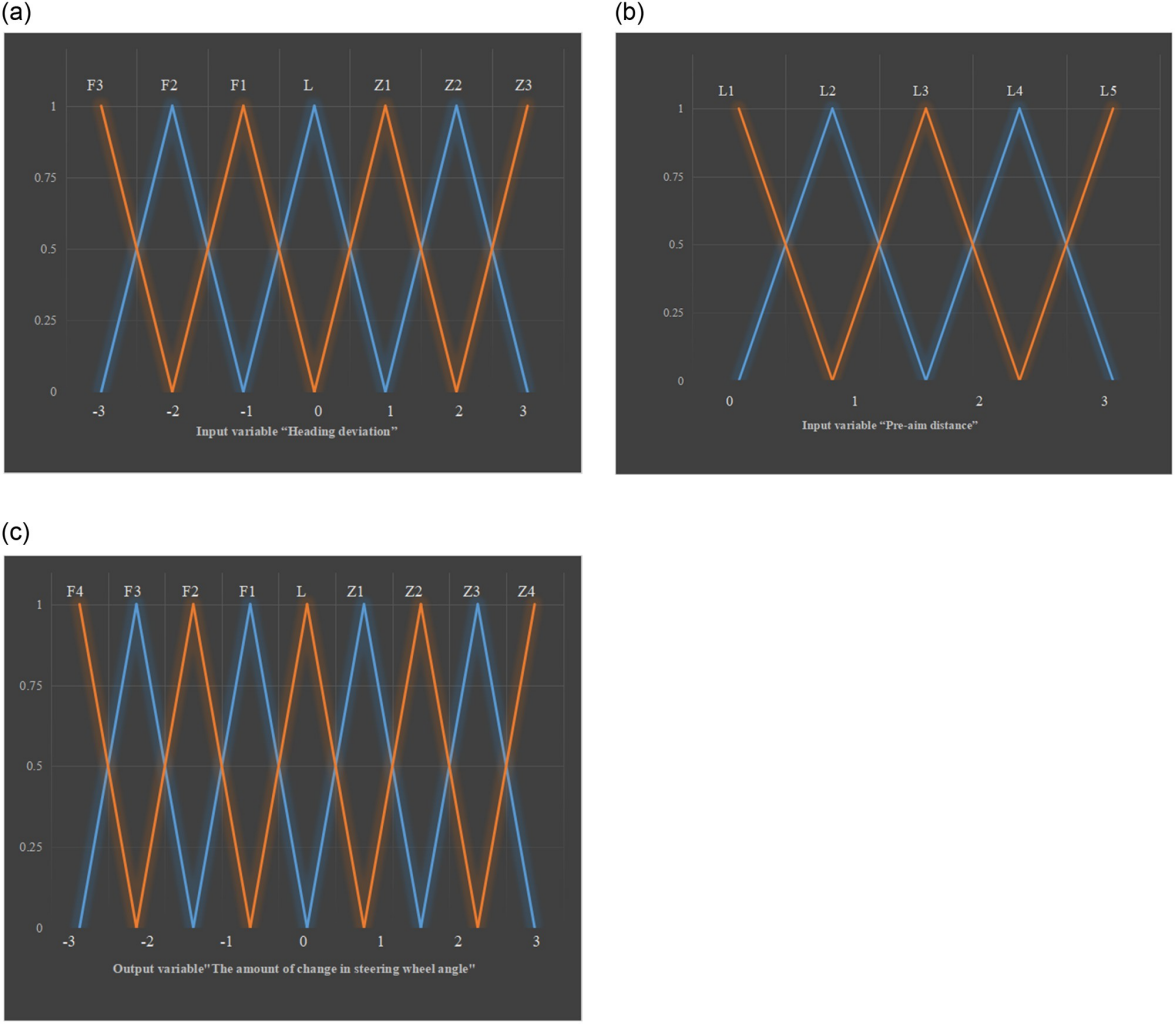
Membership function of input and output variables (a) heading deviation (b) preview distance (c) steering wheel angle change.

1. Determining the input and output variables
  The heading deviation *e*_*β*_ and preview distance *L* are used as input variables in this design; the change Δ*θ* in steering wheel angle is used as the output variable. Δ*θ* refers to the difference between the steering wheel at the current moment and the previous moment; Δ*θ* < 0 means that the steering wheel is turned to the left; Δ*θ* > 0 indicates that the steering wheel is turned to the right.
2. Variable fuzzification
  The corresponding range of the input variable *e*_*β*_ is [−30°, 30°]; the corresponding fuzzy set universe is [−3, 3]; the preview distance *L* is [0, 30], and the unit is m; the fuzzy set universe is [0, 30]. The range of the output variable steering wheel angle variation Δ*θ* is [−180°, 180°]; the fuzzy set universe is [0, 30]. Seven fuzzy subsets of heading deviation *e*_*β*_ are selected: F3, F2, F1, L, Z1, Z2, and Z3. They indicate that the target heading angle and the actual heading angle deviate a lot to the left, to the left, a little to the left, keep the same, a little to the right, a little to the right, and a lot to the right. The fuzzy subsets of preview distance *L* are S1, S2, S3, S4, and S5, meaning that the preview distance is very small, small, large, very large, and extremely large. The fuzzy subset of the output variable steering wheel angle change is F4, F3, F2, F1, L, Z1, Z2, Z3, and Z4. They demonstrate that the turn the steering wheel excessively left, left a lot, left moderately, left a little, hold still, hit a little to the right, hit the right amount, hit the right a lot, and hit the right excessively. The fuzzy subset set of input variables and output variables is expressed as:
$$e_{\beta }=\left\{F3,F2,F1,L,Z1,Z2,Z3\right\}$$ (13)
$$L=\left\{L1,L2,L3,L4,L5\right\}$$ (14)
$$\Delta \theta =\left\{F4,F3,F2,F1,L,Z1,Z2,Z3,Z4\right\}$$ (15)
  The membership function of input variable and output variable adopts triangular membership function, as shown in Fig 8.
3. Construction of fuzzy control rules
  Table 2 lists the fuzzy control rules formulated by analyzing the driver’s braking behavior during driving.
4. Clarification of output variables
  The output obtained by fuzzy rules is the fuzzy quantity, and the final output is not obtained. The area centroid method carries the corresponding fuzzy reasoning and clear calculation. The steering control system controls the steering motor auto through the output to realize the control of the braking system.

**Table 2:** Fuzzy control rules.

Steering wheel angle change		Preview distance				
		L1	L2	L3	L4	L5
Course deviation	F3	F4	F4	F3	F3	F2
	F2	F3	F3	F2	F2	F1
	F1	F3	F2	F2	F1	F1
	L	L	L	L	L	L
	Z1	Z3	Z2	Z2	Z1	Z1
	Z2	Z3	Z3	Z2	Z2	Z1
	Z3	Z4	Z4	Z3	Z3	Z2

### Design of the anti-collision system based on deep learning

The primary function of the convolutional neural network (CNN) is to process grid-structured data. CNN refers to a neural network in which the conventional matrix multiplication operation is replaced by a convolution operation [23]. In the deep neural network algorithm, the CNN mainly consists of the local receptive field, shared weight, and pooling layer. Convolution represents the mathematical operation of two real variable functions, as presented in Eq (16).


$$s\left(t\right)=\left(x*\omega \right)\left(t\right)$$ (16)

In Eq (16), the input represents the first parameter, marked as a function *x*. The second function represents the kernel function, expressed as *ω*. The output result is usually called a feature map.

**Fig 9:**
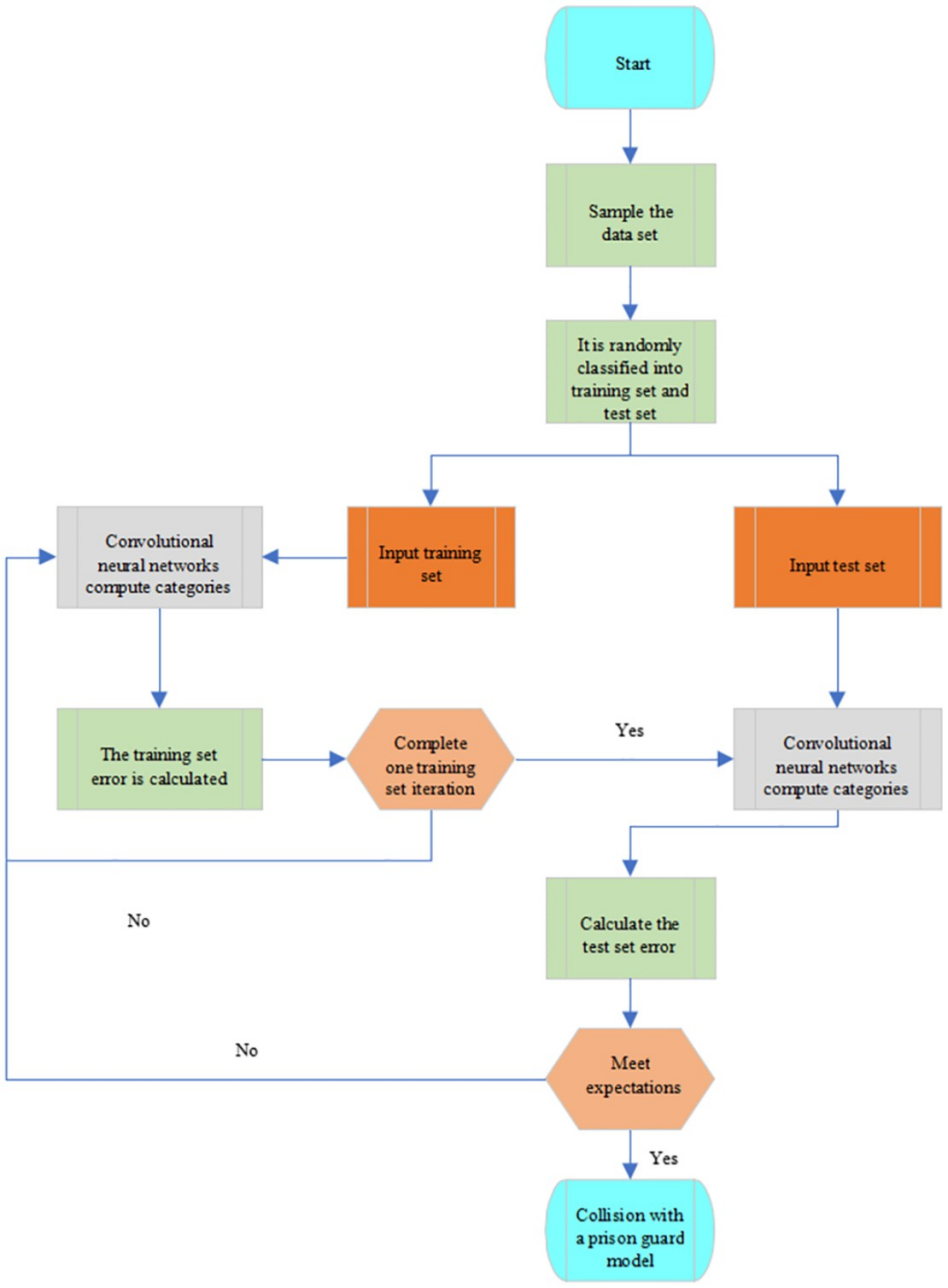
Flow of the CAW algorithm.

Reasonable system early warning plays a critical role in the safe driving of uncrewed vehicles. The early warning system, after activation, provides alarm signals to the control system to make reasonable decisions. CNN is utilized to study collision avoidance warnings and train the constructed model on accurate orbit datasets. The Collision Avoidance Warning (CAW) [24] algorithm can process the vehicle motion trajectory information to obtain and preprocess the characteristics of the collision accident and finally classify the acquired image information to complete the collision avoidance. Fig 9 shows the collision process.

The anti-collision warning model is trained by integrating the sample collection algorithm [25] and the forward learning algorithm [26]. The training set and the test set corresponding to the input model are *S*_*train*_ and *S*_*test*_, respectively. *M* indicates the output-optimized anti-collision warning model. The specific process is to initialize the CNN, then divide the sample data and input it into the neural network for training, and finally obtain the network structure. During the training process, the network parameters are continuously adjusted until the data set is traversed or the maximum number of iterations is reached to end the training.

## Results and discussion

### Optimization of the data transmission algorithm based on edge computing

The actual running time and actual accuracy of the two heuristics are evaluated to verify the practicability of the bias detection algorithm and the greedy algorithm. Table 3 lists the actual accuracy of the deviation detection algorithm and the greedy algorithm after optimization.

**Table 3:** Accuracy of two heuristic algorithms.

Periods	Optimization way	GPS accuracy / %	Land Maker accuracy/%	Lidar accuracy/%
Normal duration	Deviation monitoring algorithm	97.63	97.09	98.89
	Greedy algorithm	94.52	98.68	98.91
Rush hour	Deviation monitoring algorithm	97.07	97.54	99.48
	Greedy algorithm	93.99	98.71	99.03
Night _	Deviation monitoring algorithm	91.37	95.54	97.9
	Greedy algorithm	92.71	96.74	98.98
Accuracy requirement		91	94	97

According to the data in Table 3, the two algorithms have the highest accuracy of 99.48% and the lowest accuracy of 91% in three different application environments. The calculation effect of the two algorithms in Lidar is the best. Besides, the actual accuracy of the two heuristic algorithms can meet the accuracy requirements of the three sensors. As the number of vehicles increases, the accuracy rate shows an overall upward trend. Table 4 summarizes the average time for two algorithms to perform optimization calculations on three types of sensor data.

**Table 4:** Running time of two heuristic algorithms.

Period	Optimization way	GPS runtime/ms	Land Maker runtime/ms	Lidar runtime/ms
Normal duration	Deviation monitoring algorithm	7.3	8	7.6
	Greedy algorithm	6.9	6.6	7.0
Rush hour	Deviation monitoring algorithm	14.1	12.9	13.1
	Greedy algorithm	6.8	6.5	7.0
Night	Deviation monitoring algorithm	5.1	5.0	4.6
	Greedy algorithm	3.6	3.6	3.8

As shown in Table 4, the longest cumulative calculation time of the deviation detection algorithm under different calculation conditions is 14.1 ms, and the shortest is 4.6 ms; the longest calculation cumulative time of the greedy algorithm is 7.0 ms, and the fastest is 3.6 ms. Both algorithms perform better in Land Maker. Experimental results indicate that the deviation detection algorithm and the greedy algorithm require very little cumulative time to calculate the data transmission optimization for each sensor. The deviation detection algorithm and greedy algorithm can optimize data transmission in time and have high application values. At the same time, they can improve the safety and stability of the intelligent control system of uncrewed vehicles and help the development of uncrewed vehicles.

### Test results of the control system of self-driving cars

#### Circular trajectory tracking experiment

**Fig 10:**
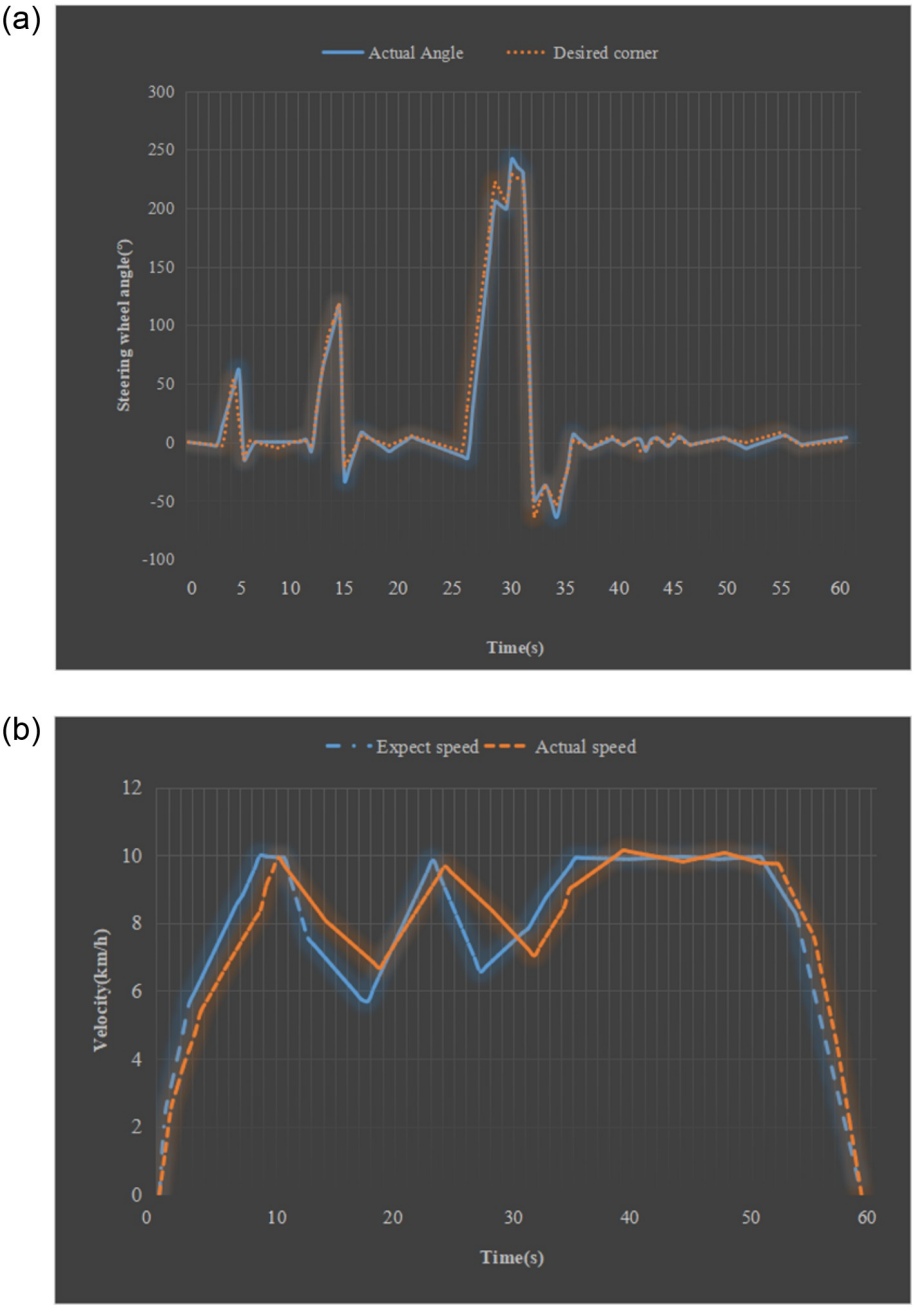
Experimental results of the vehicle control system (a) steering wheel angle curve (b) speed curve.

The steering wheel’s driving speed and turning angle during the turning process are analyzed to achieve the control effect of the vehicle control system. Fig 10 provides the response curve of the steering wheel angle.

According to Fig 10a, the effect of the lateral control algorithm gradually becomes larger in curve driving. When the vehicle turns over the target angle, the intelligent system drives the steering controller to control the steering wheel to return to the forward direction through the deviation corresponding to the driving trajectory. The steering wheel angle is constantly adjusted to become smaller and more stable. It can be found that the deviation between the two is tiny by comparing Fig 10a and 10b. The maximum deviation is only 0.7 meters during the entire curve driving process. After the vehicle completes the turn, the speed is adjusted back to the pre-planned rate through the PID algorithm. Therefore, the research method reported here can adapt well to the control requirements of current uncrewed driving technology. It can provide technical support for deep learning technology and edge computing technology to optimize uncrewed driving technology.

### Emergency braking test

**Fig 11:**
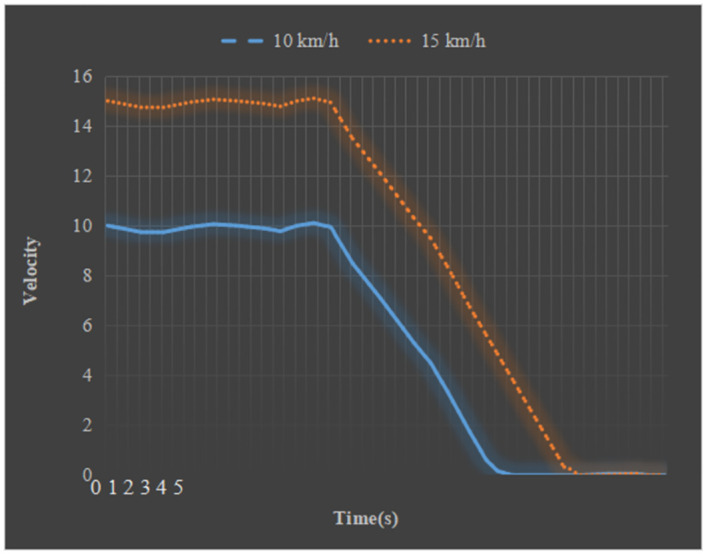
Changing curves of the emergency braking speed.

An emergency braking experiment is carried out on the experimental vehicle to verify the reliability and safety of the longitudinal control system. Car routes are planned, and obstacles are placed in pre-planned enclosed places. Fig 11 shows the speed change curve of the vehicle during the braking process.

Fig 11 shows the test process of the braking system. The experimental vehicle ran at 10km/h and 15km/h, respectively. The brake decelerated from the second round, so the experimental car’s speed dropped rapidly. During the whole braking process of the vehicle, when the initial speed is 10km/h, the deceleration process takes 1.4s, and the braking distance is 1m. The deceleration time is 2s when the initial speed is 15km/h, and the braking distance is 1.7m. The test results can generally meet the emergency braking. motion request.

### Test results of the anti-collision system based on deep learning

#### Training test results of the deep learning model

**Fig 12:**
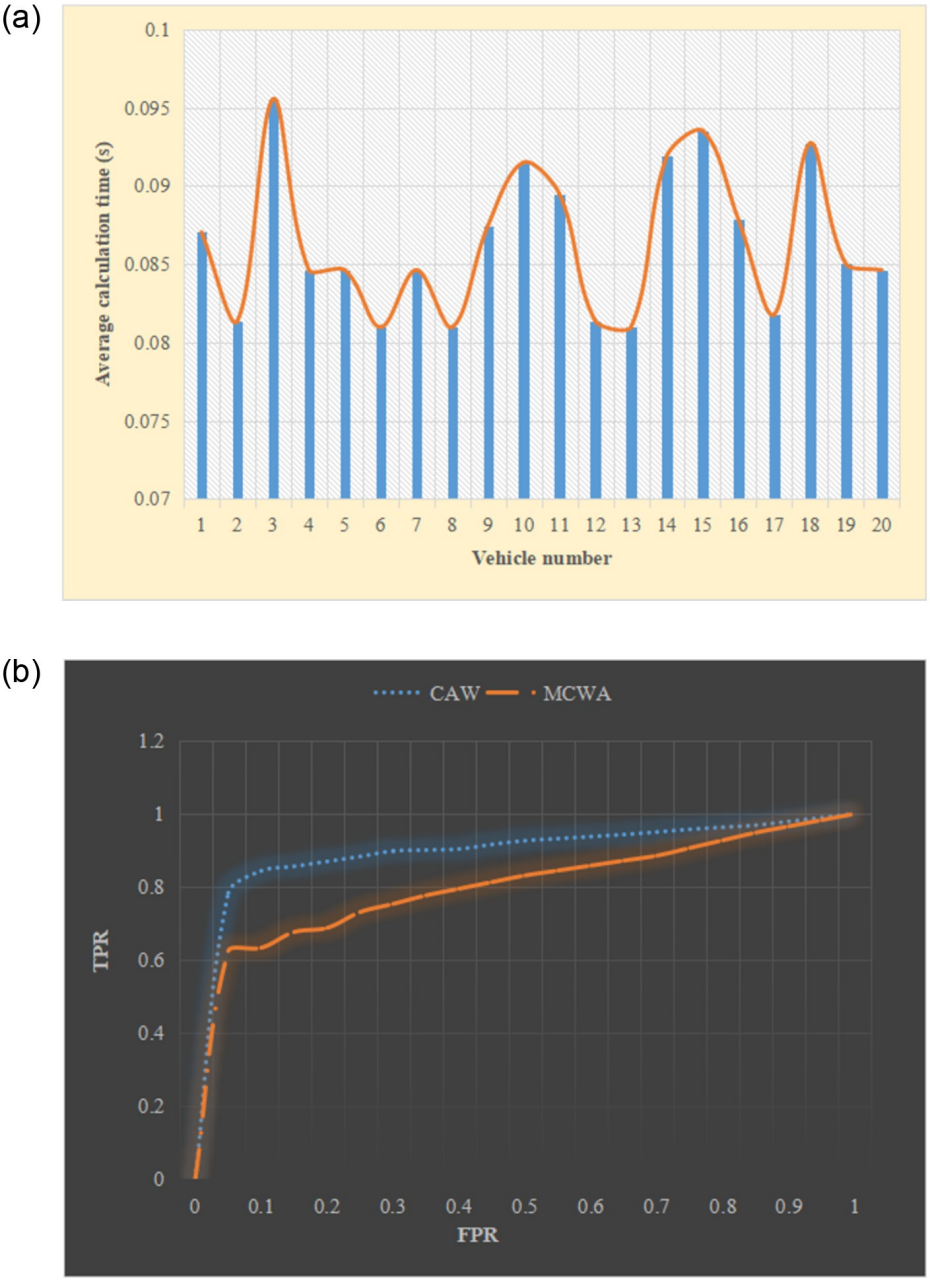
Model training results (a) Average prediction time (b) ROC curve.

MATLAB, Python, and other computer software are used to train the anti-collision model to verify its real-time performance. In the training process, some samples are selected from the sample set and input into the network to calculate the actual output *Y* and the error *D* by *D* = *B*_*i*_ − *Y*. The weight matrix *W* is adjusted according to the obtained error. The above process is repeated for all the selected samples until the specified error is reached. Twenty pieces of data were randomly selected from the example set for processing. The average prediction time and sensitivity were analyzed using the Receiver Operating Characteristic (ROC) curve. The specific situation is shown in Fig 12.

Fig 12a suggests that the error results of the 20 samples are all less than 0.1, indicating that the model has excellent real-time performance. The closer the curve in Fig 12b is to the (0, 1) point, the better the effect of the curve is. In other words, the smaller the area on the curve, the better the curve algorithm. It is found that the effect of the CAW curve is significantly better than that of the MCWA curve by comparing the ROC curve.

### Simulation test results of the anti-collision system

**Fig 13:**
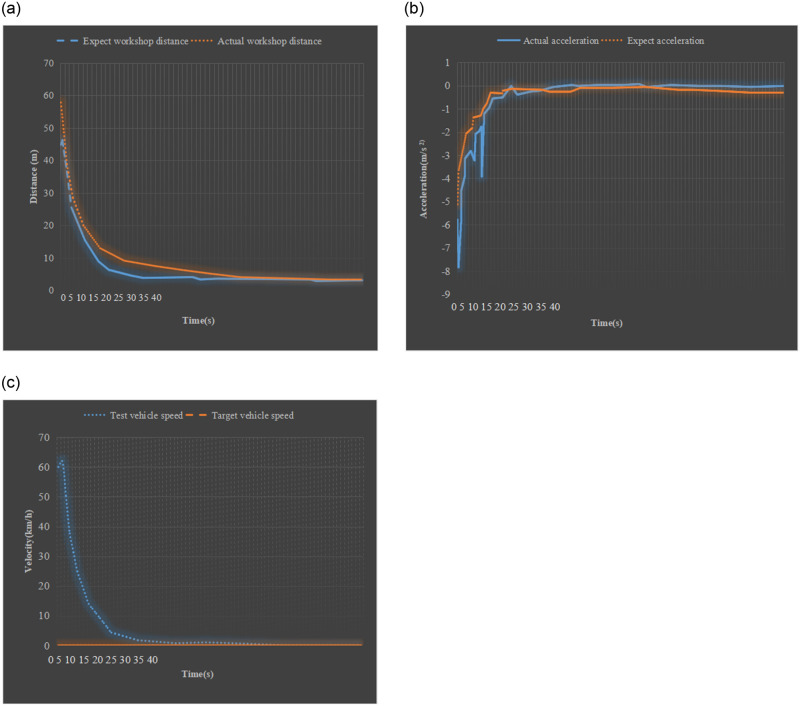
Simulation results of anti-collision of front vehicle at rest (a) comparison between actual distance and expected distance (b) comparison between actual acceleration and expected acceleration (c) comparison of two vehicle speeds.

**Fig 14:**
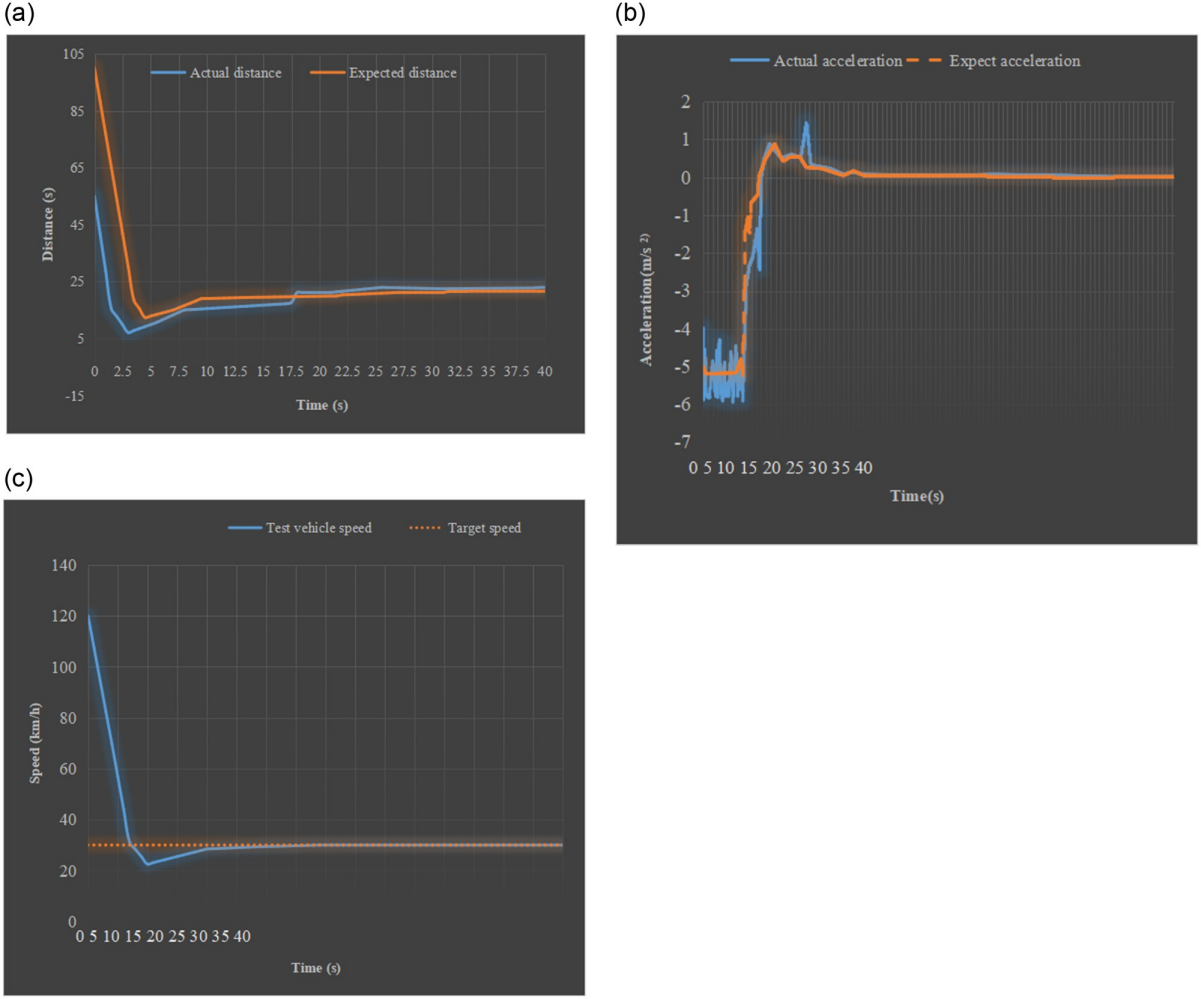
Anti-collision simulation results of the target vehicle driving at a constant speed (a) comparison between expected distance and actual distance (b) comparison between expected acceleration and actual acceleration (c) speed comparison between the two vehicles.

**Fig 15:**
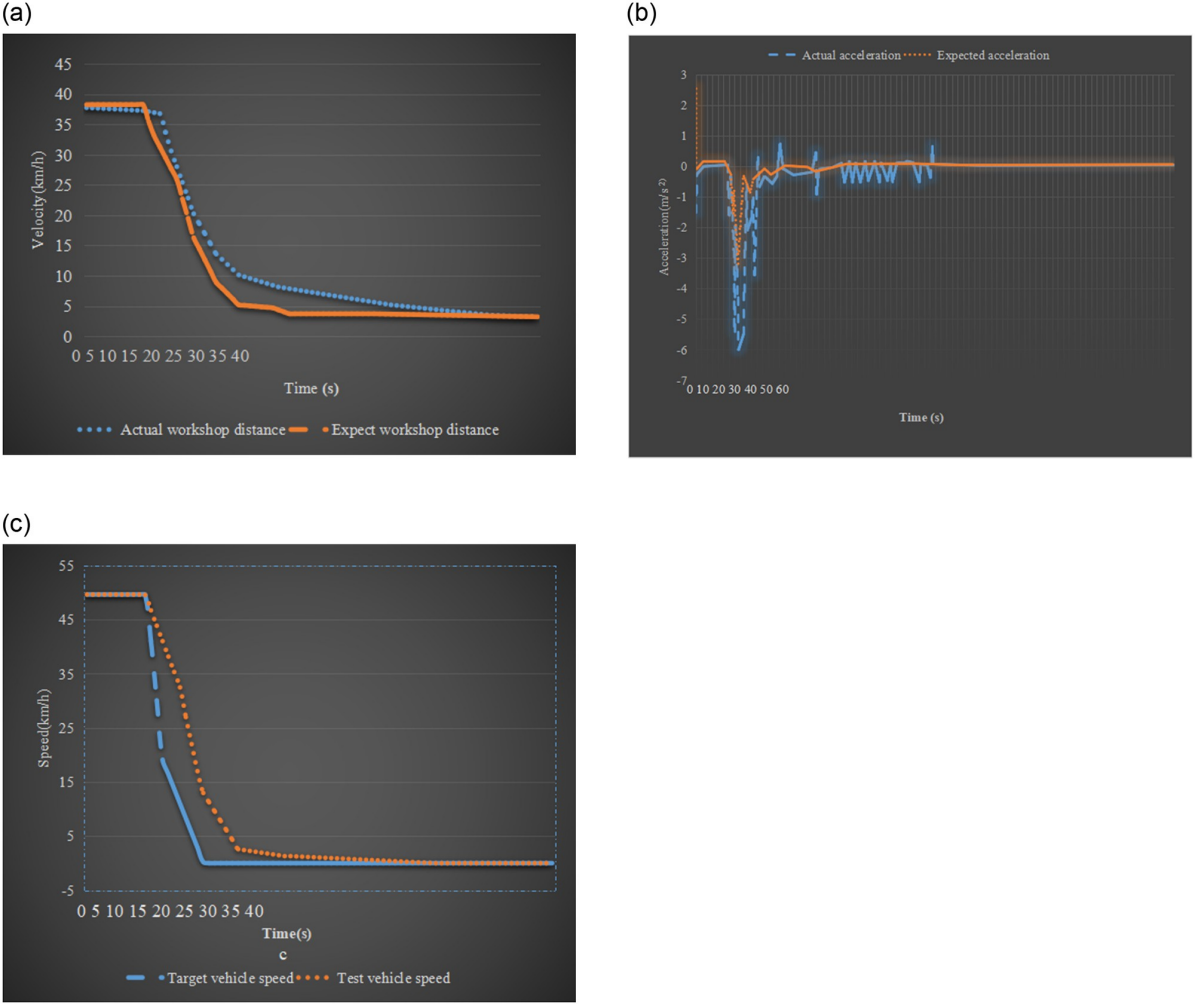
Anti-collision simulation results of the target vehicle’s emergency deceleration (a) Comparison between actual distance and expected distance (b) Comparison between actual acceleration and expected acceleration (c) Comparison of two vehicle speeds.

1. Simulation test when the vehicle ahead is stationary
  During the simulation test, the driving speed of the experimental vehicle is set to 60km/h, and the distance between the experimental vehicle and the target vehicle is 60m. Fig 13 presents the specific test results when the selected target vehicle is stationary.
  The driving speed of the experimental vehicle is 60km/h. When it is approaching the stationary target vehicle, the experimental vehicle adopts the braking operation to decelerate. After 37 seconds, the speed of the experimental vehicle drops to 0. At this time, the distance between the experimental vehicle and the stationary target vehicle is 3.7m, and no collision event occurs.
2. Simulation test of the vehicle ahead driving at a constant speed
  In the simulation experiment, the driving speed of the experimental vehicle is set to 120km/h. The distance between the experimental vehicle and the target vehicle is 50m. The driving speed of the target vehicle in front is 30km/h. The two vehicles are driving at a uniform speed. The anti-collision test results of the experimental car are shown in Fig 14.
  From the simulation results in Fig 14, it can be seen that the experimental vehicle is running at a high speed of 120km/h. While approaching the low-speed vehicle in front, the experimental vehicle decelerates fast. Besides, the current target vehicle is running normally. In the case of decreasing speed, the distance between the experimental vehicle and the target vehicle is at a safe distance.
3. Simulation test of emergency deceleration of the vehicle ahead
  Simulation experiments were carried out for two states of medium speed and high speed to verify the effectiveness of the longitudinal anti-collision system of the experimental vehicle with the sudden deceleration of the target vehicle. The driving speed of the experimental vehicle is set to 50km/h; the distance between the experimental vehicle and the target vehicle is 40m; the target vehicle in front drives at a constant speed of 50km/h. Before the 5th second, the target vehicle suddenly decelerates, and the speed drops to 0 after 5 seconds. Fig 15 illustrates the test results of the experimental vehicle anti-collision system.

In the initial stage of the above process, the actual distance between the experimental vehicle and the target vehicle is very close to the expected safe distance. Besides, the experimental vehicle is in a safe state during this process. When the experimental vehicle starts to decelerate, the experimental vehicle adopts an emergency deceleration to ensure a safe distance between the two vehicles. At this time, the car enters the second-level warning state, but the safety of driving can still be ensured. It is advisable to maintain low-speed emergency braking in this state.

## Conclusion

With science and technology’s continuous development and progress, industrial production technology is constantly optimized. Therefore, self-driving cars have emerged as required to efficiently meet society’s needs. At the same time, self-driving cars have become one of the leading directions for the future development of the automotive industry and an essential part of the intelligent transportation system. However, AI technology in self-driving cars is still not mature enough. Therefore, this study mainly studies the smart control system of driverless cars through the lateral control and longitudinal control systems of driverless cars. First, the lateral and longitudinal control systems are designed for driverless cars. Then, the steering, brake, and throttle systems are modified based on the controller designed here. In addition, deep learning models are applied to collision avoidance systems. At the same time, edge computing is used in the intelligent control system of automobiles. Finally, circular trajectory tracking and emergency braking experiments are carried out on the modified experimental vehicle to verify the practicability and reliability of the control system. The results suggest that the driving speed of the experimental vehicle is set to 50km/h; the distance between the experimental vehicle and the target vehicle is 40m; the target vehicle in front travels at a constant speed of 50km/h. The target vehicle in front of the car suddenly decelerates in 5 seconds, and the speed drops to 0 after 5 seconds. The actual distance between the experimental and target vehicles is very close to the expected safe distance. Besides, the experimental vehicle is in a safe state during this process. When the front vehicle starts decelerating, the experimental vehicle adopts emergency deceleration to ensure a safe distance between the two vehicles. The car enters the second-level early warning state at this time, but driving safety can still be guaranteed. It is advisable to maintain low-speed emergency braking in this state. Although this study conducts virtual research on the application effect of AI technology in uncrewed driving technology, it does not explore its actual application effect. Therefore, future research will comprehensively verify AI’ practical application to promote the joint development of AI technology and driverless technology.

## Supporting information

S1 Data (ZIP)
