# Supplementary material for: Exploration of the intelligent control system of autonomous vehicles based on edge computing
Source: PLoS One. 2023 Feb 2;18(2):e0281294. doi: 10.1371/journal.pone.0281294 (PMC9894409; doi:10.1371/journal.pone.0281294)
Supplement: S1 Data — (ZIP) [file pone.0281294.s001.zip › ╩2╛▌░n/Figure 1.pptx]

## Slide 1
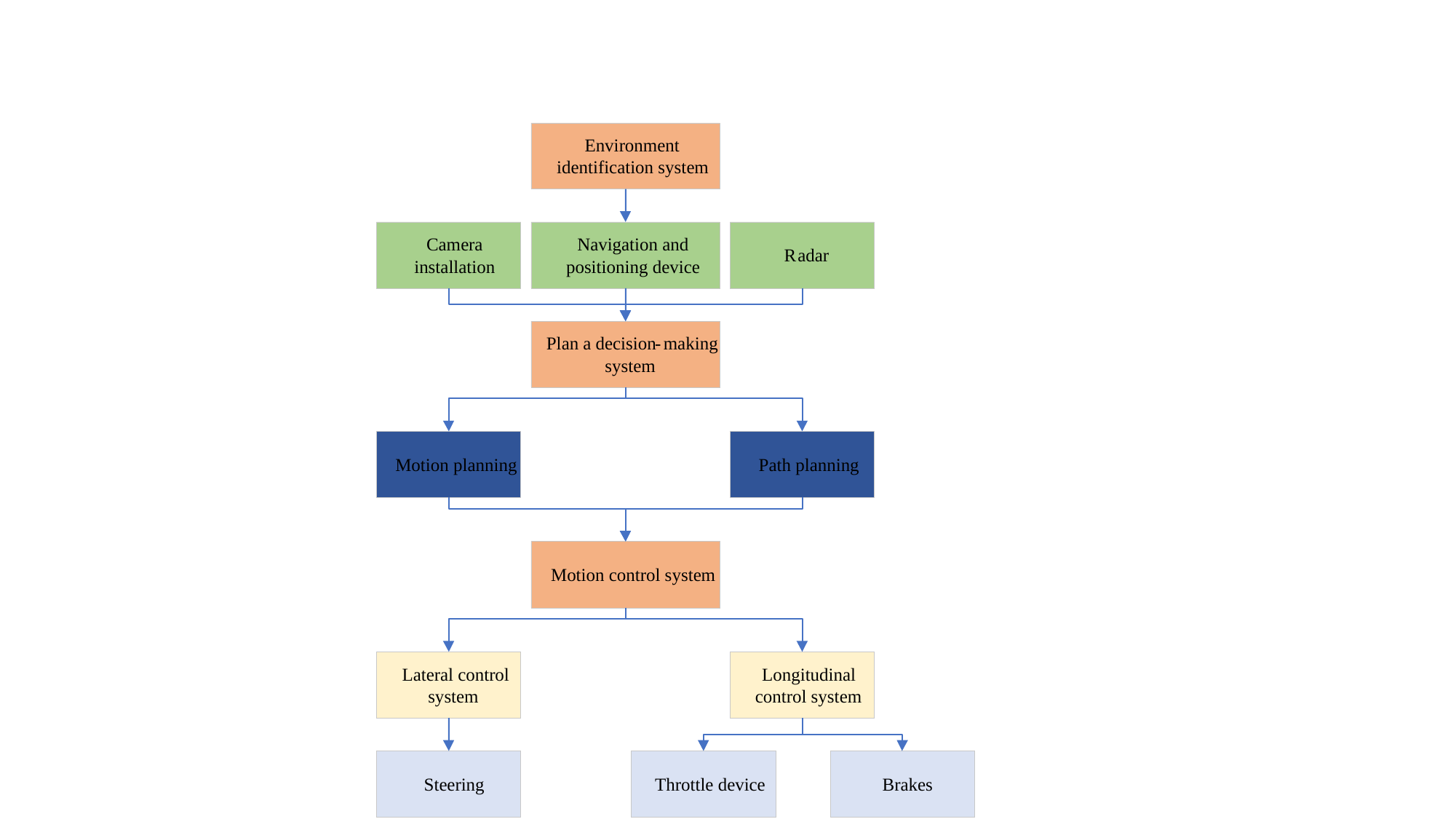

Environment
identification system
Camera
Navigation and
R
adar
installation
positioning device
Plan a decision
-
making
system
Motion planning
Path planning
Motion control system
Lateral control
Longitudinal
system
control system
Steering
Throttle device
Brakes
