# Supplementary material for: Exploration of the intelligent control system of autonomous vehicles based on edge computing
Source: PLoS One. 2023 Feb 2;18(2):e0281294. doi: 10.1371/journal.pone.0281294 (PMC9894409; doi:10.1371/journal.pone.0281294)
Supplement: S1 Data — (ZIP) [file pone.0281294.s001.zip › ╩2╛▌░n/Figure 3.pptx]

## Slide 1
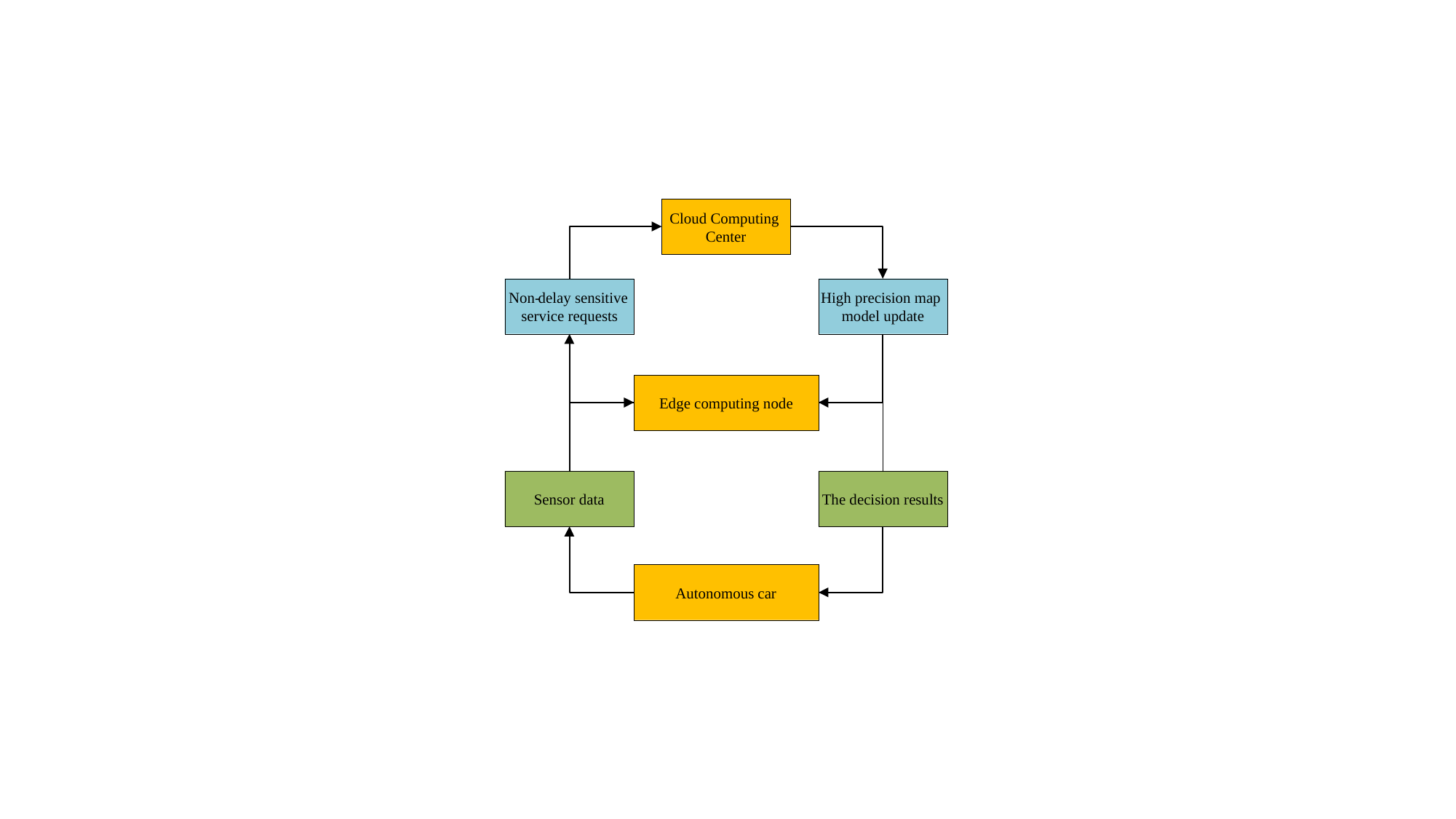

Cloud Computing
Center
Non
-
delay sensitive
High precision map
service requests
model update
Edge computing node
Sensor data
The decision results
Autonomous car
