# Supplementary material for: Exploration of the intelligent control system of autonomous vehicles based on edge computing
Source: PLoS One. 2023 Feb 2;18(2):e0281294. doi: 10.1371/journal.pone.0281294 (PMC9894409; doi:10.1371/journal.pone.0281294)
Supplement: S1 Data — (ZIP) [file pone.0281294.s001.zip › ╩2╛▌░n/Figure 4.pptx]

## Slide 1
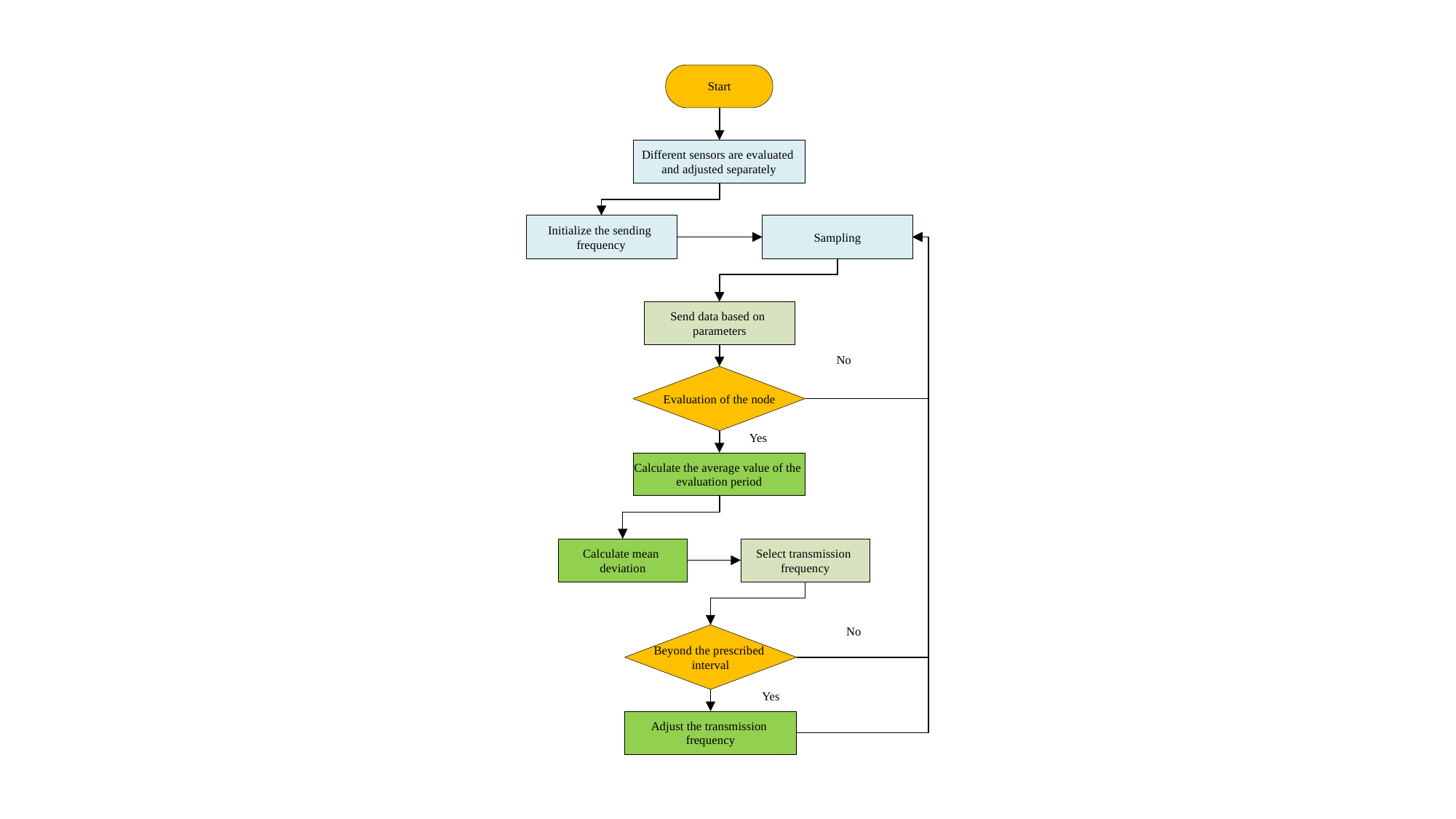

Start
Different sensors are evaluated
and adjusted separately
Initialize the sending
Sampling
frequency
Send data based on
parameters
No
Evaluation of the node
Yes
Calculate the average value of the
evaluation period
Calculate mean
Select transmission
deviation
frequency
No
Beyond the prescribed
interval
Yes
Adjust the transmission
frequency
