# Supplementary material for: Exploration of the intelligent control system of autonomous vehicles based on edge computing
Source: PLoS One. 2023 Feb 2;18(2):e0281294. doi: 10.1371/journal.pone.0281294 (PMC9894409; doi:10.1371/journal.pone.0281294)
Supplement: S1 Data — (ZIP) [file pone.0281294.s001.zip › ╩2╛▌░n/Figure 5.pptx]

## Slide 1
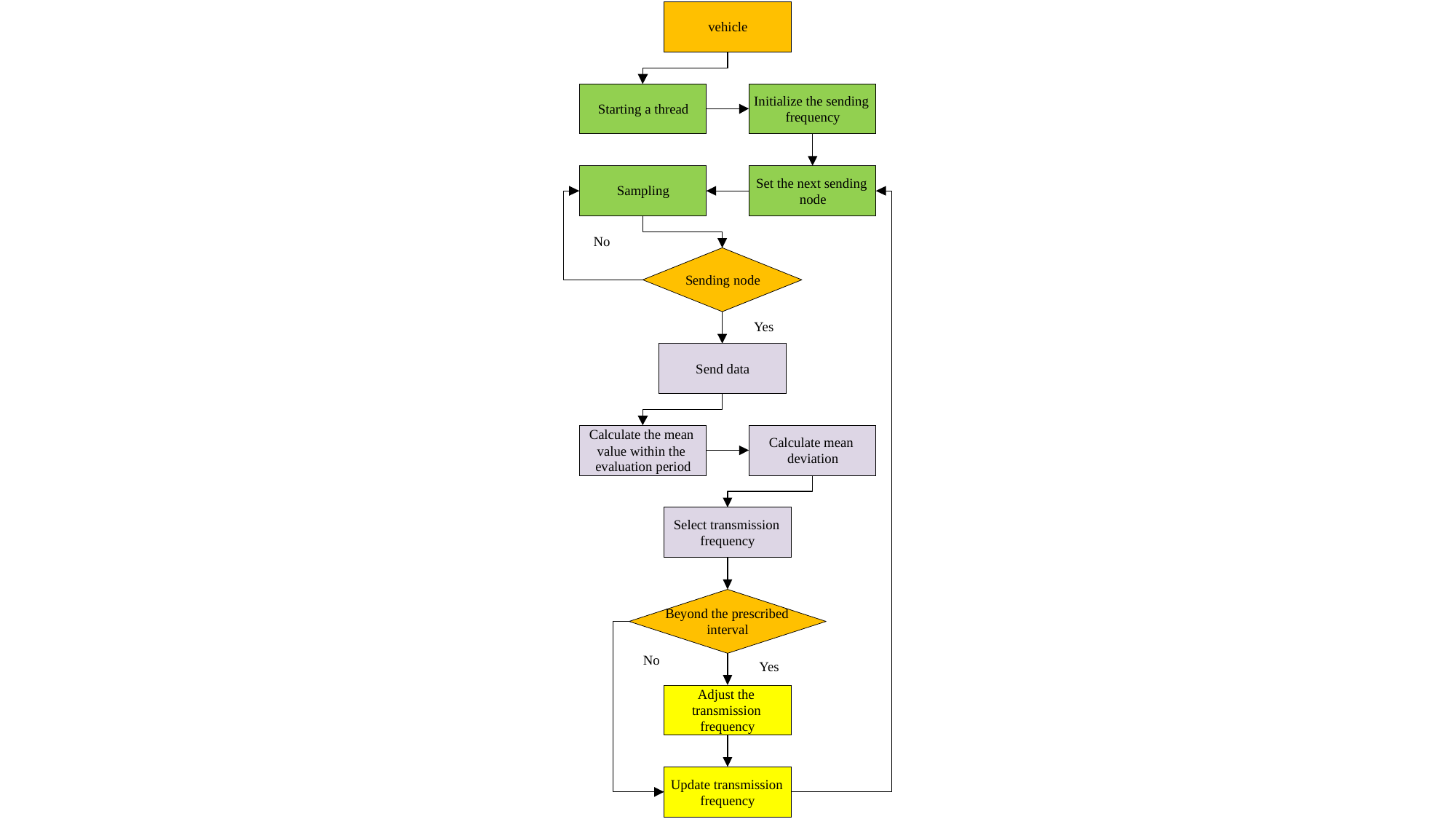

vehicle
Initialize the sending
Starting a thread
frequency
Set the next sending
Sampling
node
No
S
ending node
Yes
S
end data
Calculate the mean
Calculate mean
value within the
deviation
evaluation period
Select transmission
frequency
Beyond the prescribed
interval
No
Yes
Adjust the
transmission
frequency
Update transmission
frequency
