# Supplementary material for: Exploration of the intelligent control system of autonomous vehicles based on edge computing
Source: PLoS One. 2023 Feb 2;18(2):e0281294. doi: 10.1371/journal.pone.0281294 (PMC9894409; doi:10.1371/journal.pone.0281294)
Supplement: S1 Data — (ZIP) [file pone.0281294.s001.zip › ╩2╛▌░n/Figure 7.pptx]

## Slide 1
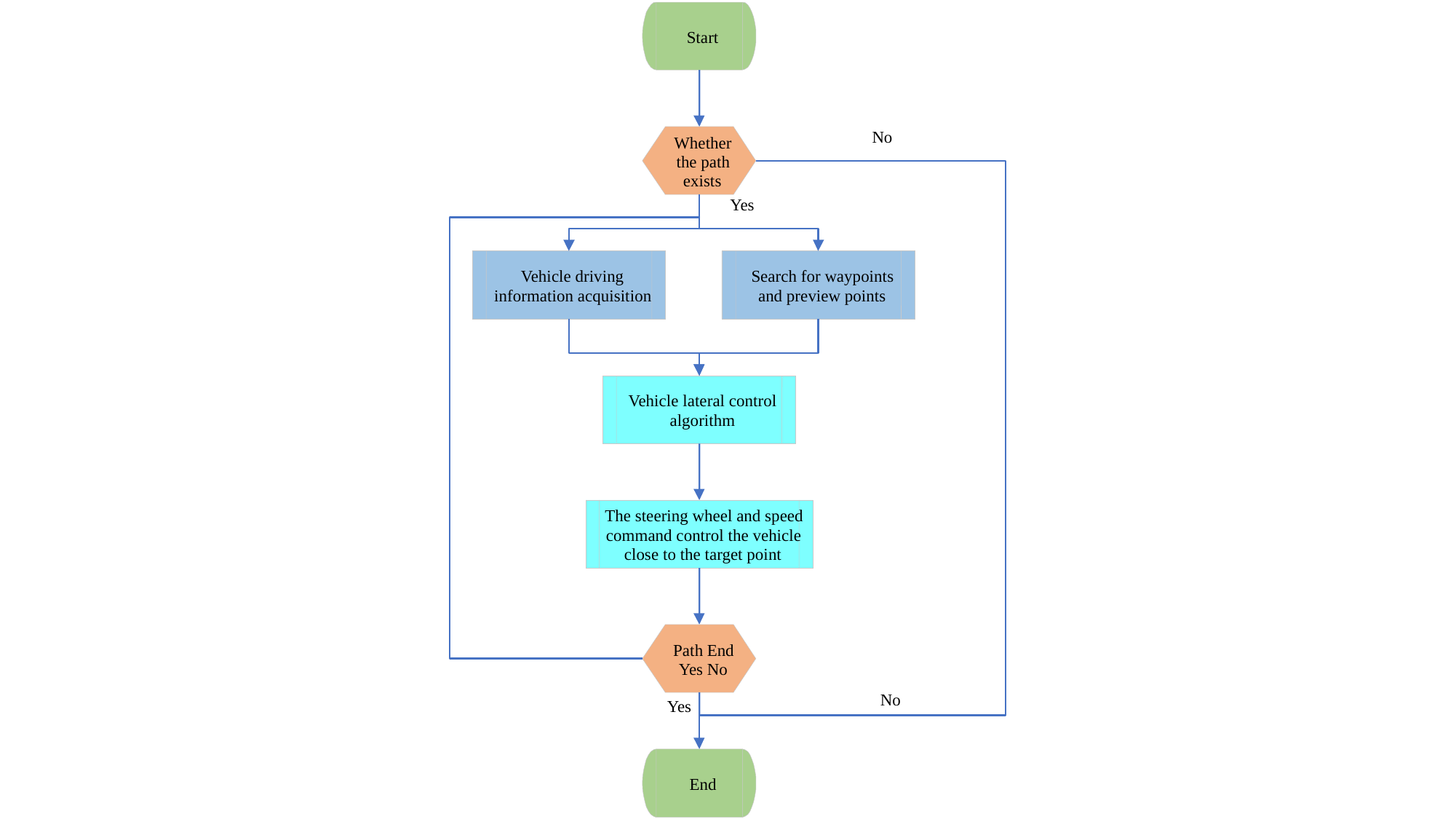

Start
No
Whether
the path
exists
Yes
Vehicle driving
Search for waypoints
information acquisition
and preview points
Vehicle lateral control
algorithm
The steering wheel and speed
command control the vehicle
close to the target point
Path End
Yes No
No
Yes
End
