# Supplementary material for: Exploration of the intelligent control system of autonomous vehicles based on edge computing
Source: PLoS One. 2023 Feb 2;18(2):e0281294. doi: 10.1371/journal.pone.0281294 (PMC9894409; doi:10.1371/journal.pone.0281294)
Supplement: S1 Data — (ZIP) [file pone.0281294.s001.zip › ╩2╛▌░n/Figure 9.pptx]

## Slide 1
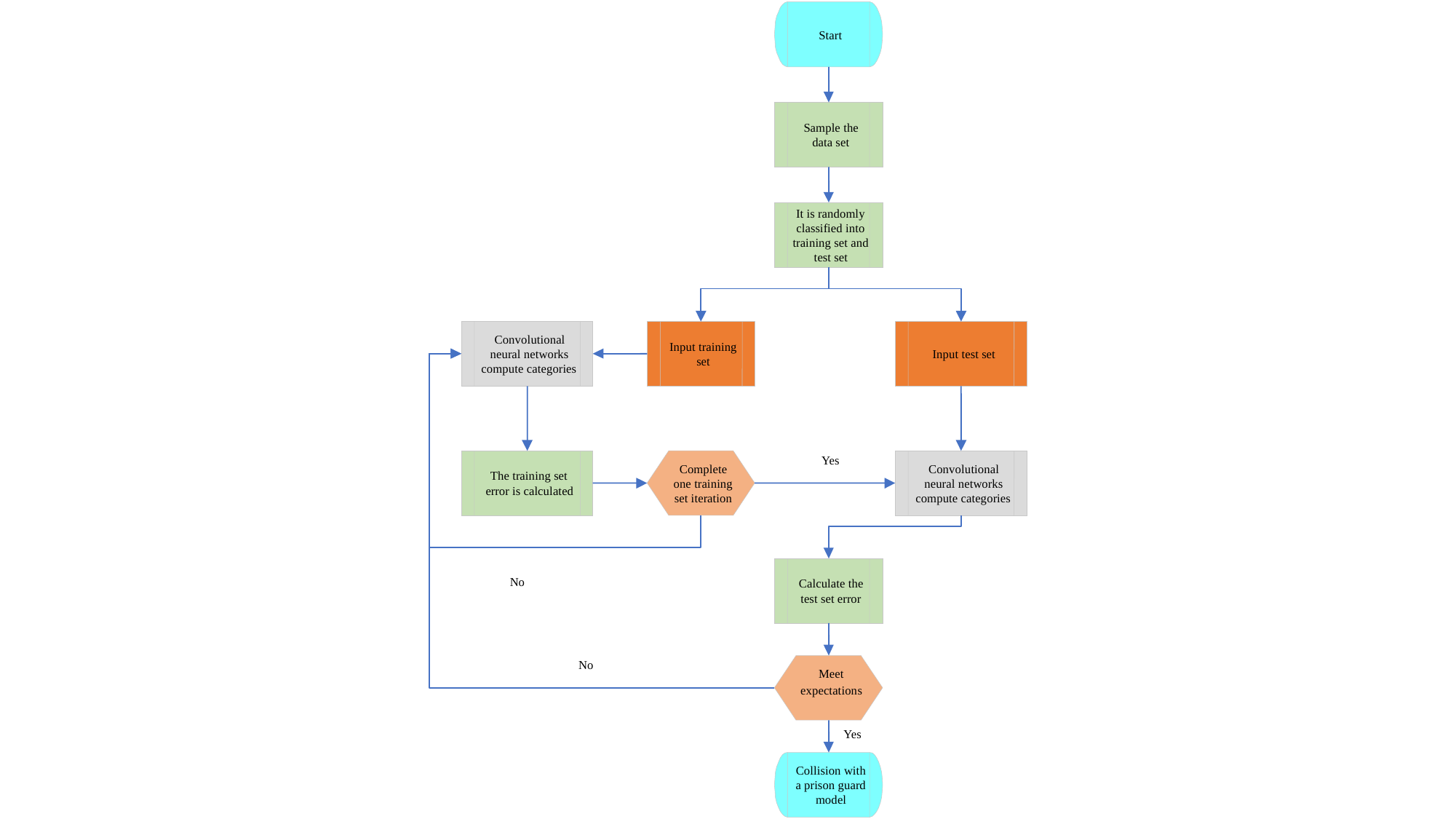

Start
Sample the
data set
It is randomly
classified into
training set and
test set
Convolutional
Input training
neural networks
Input test set
set
compute categories
Yes
Complete
Convolutional
The training set
one training
neural networks
error is calculated
set iteration
compute categories
No
Calculate the
test set error
No
Meet
expectations
Yes
Collision with
a prison guard
model
