# Supplementary figures and images for: Exploration of the intelligent control system of autonomous vehicles based on edge computing
Source: PLoS One. 2023 Feb 2;18(2):e0281294. doi: 10.1371/journal.pone.0281294 (PMC9894409; doi:10.1371/journal.pone.0281294)

## Slide 1
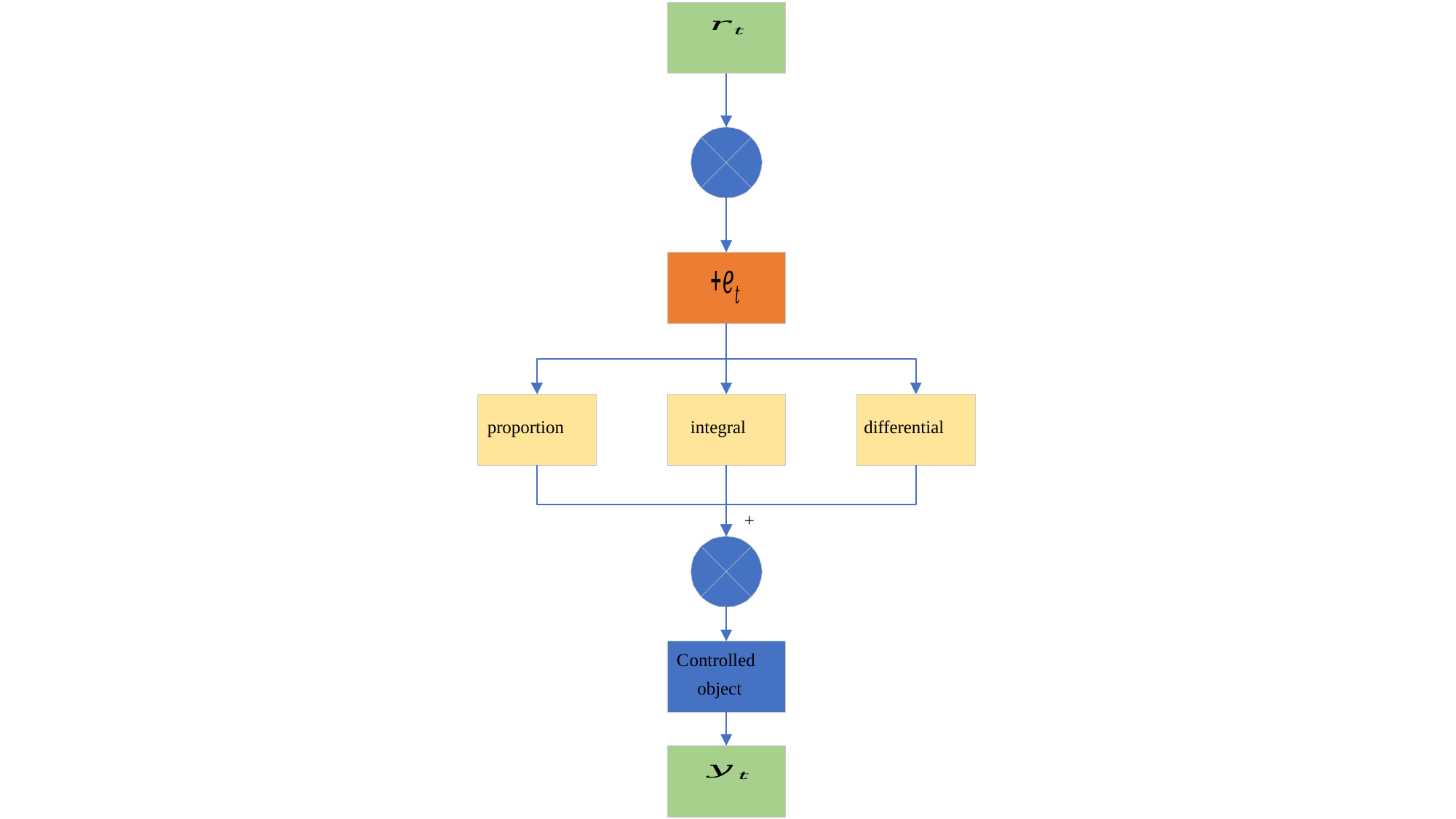

proportion
integral
differential
+
C
ontrolled
object

Supplement: S1 Data — (ZIP) [file pone.0281294.s001.zip › ╩2╛▌░n/Figure 2.pptx]
